# Adipose tissue transcriptomic signature highlights the pathological relevance of extracellular matrix in human obesity

**DOI:** 10.1186/gb-2008-9-1-r14

**Published:** 2008-01-21

**Authors:** Corneliu Henegar, Joan Tordjman, Vincent Achard, Danièle Lacasa, Isabelle Cremer, Michèle Guerre-Millo, Christine Poitou, Arnaud Basdevant, Vladimir Stich, Nathalie Viguerie, Dominique Langin, Pierre Bedossa, Jean-Daniel Zucker, Karine Clement

**Affiliations:** 1 INSERM, UMR-S 872, Les Cordeliers, Eq. 7 Nutriomique and Eq. 13, Paris, F-75006 France; 2 Pierre et Marie Curie-Paris 6 University, Cordeliers Research Center, UMR-S 872, Paris, F-75006 France; 3 Paris Descartes University, UMR-S 872, Paris, F-75006 France; 4 Assistance Publique-Hôpitaux de Paris (AP-HP), Pitié Salpêtrière Hospital, Nutrition and Endocrinology department, Paris, F-75013 France; 5 Franco-Czech Laboratory for Clinical Research on Obesity, INSERM and 3rd Faculty of Medicine, Charles University, Prague, CZ-10000, Czech Republic; 6 INSERM, U858, Obesity Research Laboratory, I2MR, Toulouse, F-31432 France; 7 Paul Sabatier University, Louis Bugnard Institute IFR31, Toulouse, F-31432 France; 8 Centre Hospitalier Universitaire de Toulouse, Toulouse, F-31059 France; 9 Assistance Publique-Hôpitaux de Paris (AP-HP), Beaujon Hospital, Pathology department, Clichy, F-92110 France; 10 CNRS, UMR 8149, Clichy, F-92110 France; 11 IRD UR Géodes, Centre IRD de l'Ile de France, Bondy, F-93143 France

## Abstract

**Background:**

Investigations performed in mice and humans have acknowledged obesity as a low-grade inflammatory disease. Several molecular mechanisms have been convincingly shown to be involved in activating inflammatory processes and altering cell composition in white adipose tissue (WAT). However, the overall importance of these alterations, and their long-term impact on the metabolic functions of the WAT and on its morphology, remain unclear.

**Results:**

Here, we analyzed the transcriptomic signature of the subcutaneous WAT in obese human subjects, in stable weight conditions and after weight loss following bariatric surgery. An original integrative functional genomics approach was applied to quantify relations between relevant structural and functional themes annotating differentially expressed genes in order to construct a comprehensive map of transcriptional interactions defining the obese WAT. These analyses highlighted a significant up-regulation of genes and biological themes related to extracellular matrix (ECM) constituents, including members of the integrin family, and suggested that these elements could play a major mediating role in a chain of interactions that connect local inflammatory phenomena to the alteration of WAT metabolic functions in obese subjects. Tissue and cellular investigations, driven by the analysis of transcriptional interactions, revealed an increased amount of interstitial fibrosis in obese WAT, associated with an infiltration of different types of inflammatory cells, and suggest that phenotypic alterations of human pre-adipocytes, induced by a pro-inflammatory environment, may lead to an excessive synthesis of ECM components.

**Conclusion:**

This study opens new perspectives in understanding the biology of human WAT and its pathologic changes indicative of tissue deterioration associated with the development of obesity.

## Abstract 2

Analysis of the transcriptomic signature of white adipose tissue in obese human subjects revealed increased interstitial fibrosis and an infiltration of inflammatory cells into the tissue.

## Background

Investigations performed in mice and humans have led to a pathophysiological paradigm that acknowledges obesity as a low-grade inflammatory disease. Elevated inflammatory proteins in obese individuals [1] suggest that inflammation may play a determinant role in connecting obesity to metabolic, hepatic and cardiovascular diseases [2], and to some cancers [3]. In such chronic pathologies, in which obesity appears as a well established risk factor, a prominent role for the immuno-inflammatory processes has been put forward as contributing to disease progression and tissue deterioration [4]. However, in spite of substantial evidence demonstrating the existence of a low-grade inflammatory component in obesity [5], the molecular mechanisms that link inflammatory changes to the development, aggravation, maintenance, and resistance to treatment that characterize obesity states remain poorly understood.

White adipose tissue (WAT), now considered as a pivotal endocrine organ, contributes to the systemic inflammation by producing biomolecules, including pro-inflammatory mediators, whose estimated number grows constantly and whose synthesis is altered along with the expansion of the adipose tissue [6,7]. These molecules are delivered into the blood stream and exert metabolic and immune functions, as illustrated by the extensively studied adipose hormones leptin and adiponectin. Their functions are essential for inter-organ cross-talk, body weight homeostasis and probably in linking adipose tissue to the downstream complications associated with obesity [8]. Cellular types composing WAT include mature adipocytes, the specialized metabolic cells, and a variety of other cells grouped in the 'stroma vascular fraction' (SVF), which are not well characterized in humans. Although some molecules secreted by WAT, such as leptin and adiponectin, are synthesized by mature adipocytes [8], the non-adipose SVF, comprising infiltrated macrophages among other cellular types, is a source of inflammation-related molecules that may exert a local action on adipose tissue biology, particularly within the enlarged WAT [9-11]. The possible infiltration of the obese WAT by other inflammatory cells is also suggested by recent analyses in mice showing the modulation of T and natural killer (NK) cell subtypes in animals fed with a high fat diet [12]. Adipose loss leads to the improvement of the inflammatory profile [11], with a concomitant reduction of infiltrating macrophages [13].

In obese human subjects, large-scale transcriptomic analyses of WAT, in stable weight conditions or during weight loss, led mostly to the description of inflammatory changes and produced extensive lists of regulated genes involved in a number of biological functions [14]. However, the relationship between these genes, the cellular processes in which they are involved, and the tissue structure as a whole remains poorly understood. To address this question, we took advantage of increasing progress in the analysis of complex biological interactions, which has attracted a great amount of interest in various fields. An important motivation for the study of such networks of biological interactions resides in their ability to formally characterize the roles played by various interacting elements comprising cellular environments, thus helping prioritize further mechanistic investigations. In particular, the study of gene interaction networks, constructed by relating co-expressed genes (that is, genes sharing similar expression profiles), contributed to the characterization of several key properties of biological networks, such as the scale-free distribution of their connectivity [15], their hierarchical architecture built from modules of functionally related components (that is, genes, enzymes, metabolites) [15], the various types of net hubs [16], or the small-world aspect of their fast synchronizability [17]. Along with the development of interactions analysis, the biological interpretation of large-scale gene expression profiling data has evolved gradually into a highly standardized and powerful analytical framework. Available exploratory tools rely on curated gene annotation resources and standardized statistical evaluation techniques to identify significantly over-represented biological themes in high-throughput gene expression datasets [18].

The objective of our study was to construct a full-scale map of the biological interactions defining the transcriptomic signature of WAT in obese subjects. For this purpose we devised an original analytical approach, which further extended the conventional gene co-expression network analysis to include the evaluation of transcriptomic interactions between relevant biological themes, including cellular components, biological processes and regulatory or metabolic pathways. This approach was applied to the analysis of two sets of microarray gene expression profiles obtained previously from human WAT of obese subjects in stable weight conditions [11,19] and three months after significant weight loss induced by gastric surgery [13]. Our analysis revealed major and interrelated changes of WAT transcriptomic signature in obese human subjects, involving extracellular matrix (ECM), and inflammatory and adipose metabolic processes. Tissue and cellular investigations, directed by the hypotheses raised by the analysis of gene and functional interactions, show that subcutaneous adipose tissue of obese subjects is characterized by an excessive amount of interstitial fibrosis and suggest that the phenotypic changes in human pre-adipocytes, induced by a pro-inflammatory environment, are associated with excessive synthesis of ECM components, which may contribute to tissue deterioration.

## Results

### The transcriptomic signature of the subcutaneous WAT in obese subjects

Thirty five cDNA microarray experiments were performed in 25 weight-stable obese subjects (body mass index (BMI) 40.58 ± 1.58 kg/m^2^, range 32.6-60.5 kg/m^2^) and 10 healthy lean controls (BMI 23.67 ± 0.48 kg/m^2^, range 21.4-26.2 kg/m^2^) to characterize the transcriptomic signature of the subcutaneous WAT associated with chronic obesity. The overall clinical and biochemical parameters of the studied population are presented in Table 1, and on the companion website as online supplementary data [20]. The analysis of the differential gene expression with the Significance analysis of microarrays (SAM) procedure [21], performed on the cDNA measurements with signals recovered in at least 80% of the microarray experiments, detected 366 up- and 474 down-regulated genes, corresponding to a 5% false discovery rate (FDR). The functional analysis of these genes identified 704 genes (307 up- and 397 down-regulated) annotated with Gene Ontology (GO) categories [22], and 253 genes (101 up- and 152 down-regulated) annotated with categories of the Kyoto Encyclopedia of Genes and Genomes (KEGG) [23].

**Table 1:** Overall clinical and biological parameters of 55 obese subjects and 15 lean controls

Phenotype	Obese subjects	Lean controls
n	55	15
Female/Male	52/3	15/0
Age (years)	40.13 ± 11.67	34.2 ± 8.52
BMI (kg/m^2^)	44.07 ± 9.06*	23.67 ± 1.51
**Glucose homeostasis**		
Glucose (mmol/l)	5.56 ± 1.70	4.82 ± 1.01
Insulin (μU/ml)	13.51 ± 8.57	7.20 ± 3.49
QUICKI	0.33 ± 0.05	0.36 ± 0.04
**Type 2 diabetes**		
Glycemia > 7 mmol/l or treatment	6 (11%)	0
**Lipid homeostasis**		
Cholesterol (mmol/l)	5.22 ± 1.04	4.36 ± 1.05
HDL cholesterol (mmol/l)	1.27 ± 0.34	1.43 ± 0.23
Triglycerides (mmol/l)	1.40 ± 0.62^†^	0.45 ± 0.10
**Adipokines**		
Leptin (ng/ml)	54.55 ± 19.92	11.24 ± 1.12
Adiponectin (μg/ml)	7.14 ± 2.87	-
**Risk factors**		
HDL < 1.03 mmol/l (M), < 1.29 mmol/l (F)	26 (47%)*	1 (6%)
Hypertension ≥ 130/85 mmHg	11 (20%)	0
Glucose ≥ 5.6 mmol/l	17 (31%)	1 (6%)
Triglycerides ≥ 1.7 mmol/l	11 (20%)	0
**Inflammatory factors**		
TNF-α (pg/ml)	1.77 ± 0.62	-
IL6 (pg/ml)	2.24 ± 1.17	-
hsCRP (mg/dl)	8.62 ± 10.38	-
Orosomucoid (g/l)	0.99 ± 0.18	-
Serum amyloid A (μg/ml)	21.35 ± 22.39	-
**Hepatic factors**		
Aspartate aminotransferase (IU/l)	22.66 ± 6.87	-
Alanine aminotransferase (IU/l)	35.72 ± 18.56	-
γGT (mg/dl)	45.91 ± 46.43	-

*Bilateral significance *p *value < 0.05 for the difference between the two groups. ^†^Bilateral significance *p *value < 0.001 for the difference between the two groups. Hyphens indicate parameters that were not available for the lean controls group. F, female; HDL, high-density lipoproteins; M, male.

**Figure 1:**
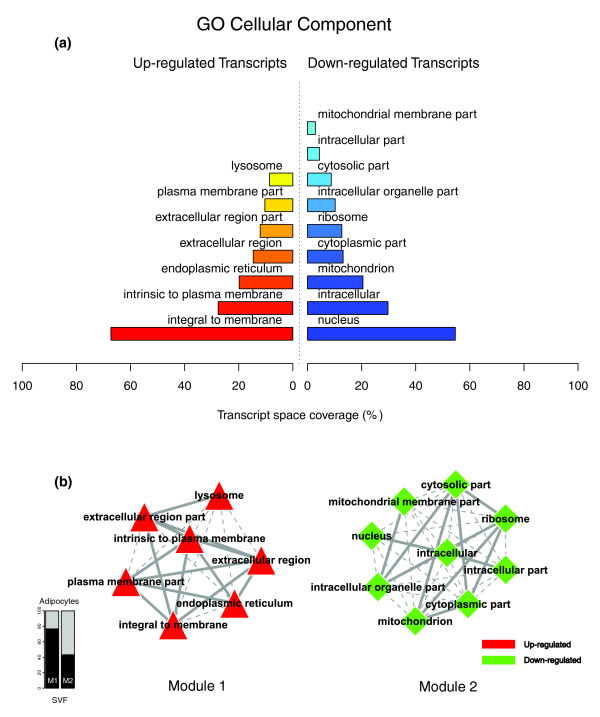
GO Cellular Component enriched themes and their interaction map, illustrating the transcriptomic signature of obese WAT. **(a) **The GO Cellular Component annotation categories showing a significant enrichment in genes up- or down-regulated in WAT of obese subjects. **(b) **These categories were related to construct a biological interaction map after quantifying their proximity based on the expression similarity of their annotated genes. Continuous lines indicate the strongest interactions (that is, superior to the upper quartile of their distribution), while dashed lines depict medium strength interactions (that is, superior to the median of the distribution but inferior to its upper quartile). The enrichment in genes expressed preferentially in one of the two main cellular fractions of WAT, illustrated in a percentage scale (mature adipocytes in light gray versus SVF in black), was significantly different in the two modules (*p *value < 0.001).

**Figure 2:**
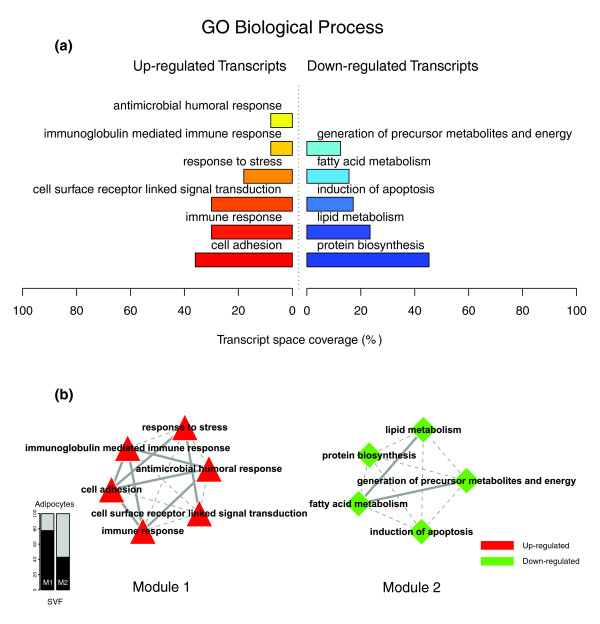
GO Biological Process enriched themes and their interaction map, illustrating the transcriptomic signature of obese WAT. **(a) **The GO Biological Process annotation categories showing a significant enrichment in genes up- or down-regulated in WAT of obese subjects. **(b) **These categories were related to construct a functional interaction map after quantifying their proximity based on the expression similarity of their annotated genes. Continuous lines indicate the strongest interactions (that is, superior to the upper quartile of their distribution), while dashed lines depict medium strength interactions (that is, superior to the median of the distribution but inferior to its upper quartile). The enrichment in genes expressed preferentially in one of the two main cellular fractions of WAT, illustrated in a percentage scale (mature adipocytes in light gray versus SVF in black), was significantly different in the two modules (*p *value < 0.05).

**Figure 3:**
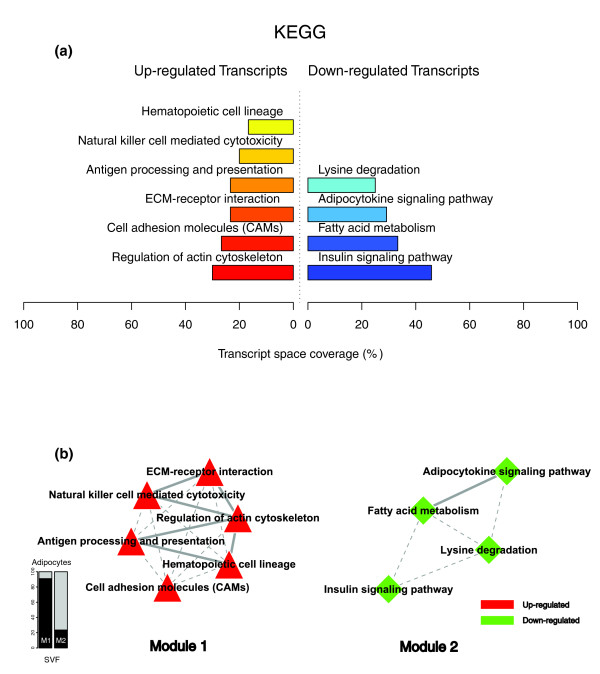
KEGG enriched themes and their interaction map, illustrating the transcriptomic signature of obese WAT. **(a) **The KEGG annotating categories showing a significant enrichment in genes up- or down-regulated in the WAT of obese subjects. **(b) **These categories were related to construct a functional interaction map after quantifying their proximity based on the expression similarity of their annotated genes. Continuous lines indicate the strongest interactions (that is, superior to the upper quartile of their distribution), while dashed lines depict medium strength interactions (that is, superior to the median of the distribution but inferior to its upper quartile). The enrichment in genes expressed preferentially in one of the two main cellular fractions of WAT, illustrated in a percentage scale (mature adipocytes in light gray versus SVF in black), was significantly different in the two modules (*p *value < 0.001).

Figures 1a, 2a and 3a illustrate the biological themes characterizing the transcriptomic signature of the subcutaneous WAT in obese subjects. Relevant biological themes, annotating genes differentially expressed in the obese WAT compared to lean controls, are indicated by significantly over-represented categories from the GO Cellular Component and Biological Process ontologies and from KEGG. While the genes up-regulated in the obese WAT were annotated mainly by structural and functional themes associated with the cellular membrane and the extracellular space, the down-regulated genes were annotated mostly by themes related to the intracellular domain. We relied on our in-house analytical approach to quantify transcriptomic interactions between these themes by aggregating the similarities of their annotated gene expression profiles (see Materials and methods for details), and then related them to build biological interaction maps. This analysis uncovered a highly segregated transcriptomic interaction pattern, regardless of the system used to annotate differentially expressed genes (Figures 1b, 2b and 3b). Two distinct types of biological interaction modules (indicated hereafter as module 1 and module 2) have been identified, one associating structural components, processes and regulatory pathways related to cellular membranes and the extracellular space (module 1), while the other groups components, processes and pathways associated with the intracellular domain (module 2).

GO Cellular Component categories annotating up-regulated genes (Figure 1a) formed a first module (Figure 1b, module 1) composed from themes primarily related to membrane components ('integral to membrane', 'plasma membrane part', 'intrinsic to plasma membrane') and to the extracellular region ('extracellular region', 'extracellular region part'). 'Lysosome' and 'endoplasmic reticulum' were the only categories designating intracellular organelles in this module. The biological processes designated by GO Biological Process categories annotating up-regulated genes (Figure 2a,b) were related to immune, inflammatory, and stress responses ('immunoglobulin mediated immune response', 'antimicrobial humoral response', 'immune response', 'response to stress'), as well as to cell adhesion and signaling processes ('cell adhesion', 'cell surface receptor linked signal transduction'). The KEGG pathways annotating genes up-regulated in the obese WAT (Figure 3a) formed a strong interaction module associating categories related to immunological and inflammatory responses as well as to cellular adhesion and signaling mechanisms (Figure 3b, module 1).

A very distinctive biological pattern was observed for themes associated with the genes down-regulated in the obese WAT. GO Cellular Component structural categories annotating these genes (Figure 1a) formed a second module (Figure 1b, module 2), grouping themes associated with intracellular components, among which are the nucleus, the cytoplasm, the ribosome and the mitochondrion ('intracellular', 'nucleus', 'cytoplasmic part', 'ribosome', 'intracellular organelle part', 'cytosolic part', 'intracellular part', 'mitochondrion', 'mitochondrial membrane part'). GO Biological Process categories annotating down-regulated genes (Figure 2a,b) were essentially related to lipid, protein and energy metabolism ('lipid metabolism', 'fatty acid metabolism', 'protein biosynthesis', 'generation of precursor metabolites and energy'), as well as to the regulation of the apoptotic machinery ('induction of apoptosis'). The examination of KEGG pathways revealed a similar interaction pattern associating a number of key adipocyte metabolic and regulatory pathways (Figure 3a,b, module 2)

Since the analysis of transcriptomic interactions in the obese WAT revealed a neat segregated pattern, we sought to determine the tissular fraction specificity of the two types of interaction modules. Taking advantage of our previous large-scale transcriptomic analysis [11], we explored the specific enrichment of isolated WAT cellular fractions in genes annotated with categories belonging to one of the two types of modules. This analysis showed that biological themes related to the extracellular space (module 1) were annotating genes predominantly expressed in the SVF of WAT, while the genes annotated with themes related to the intracellular domain (module 2) were expressed predominantly in mature adipocytes (Figures 1b, 2b and 3b).

#### ECM remodeling and inflammation related genes

We then examined the similarity between the expression profiles of individual genes to build the co-expression network underlying the described functional interactions. Among the genes annotated with significantly over-represented GO categories, 40 genes (12.5%, among which 24 genes were up-regulated and 16 genes down-regulated) were found to encode various structural components of the ECM or molecules involved in ECM remodeling and regulation (Additional data file 2 and supplementary online table 1).

**Figure 4:**
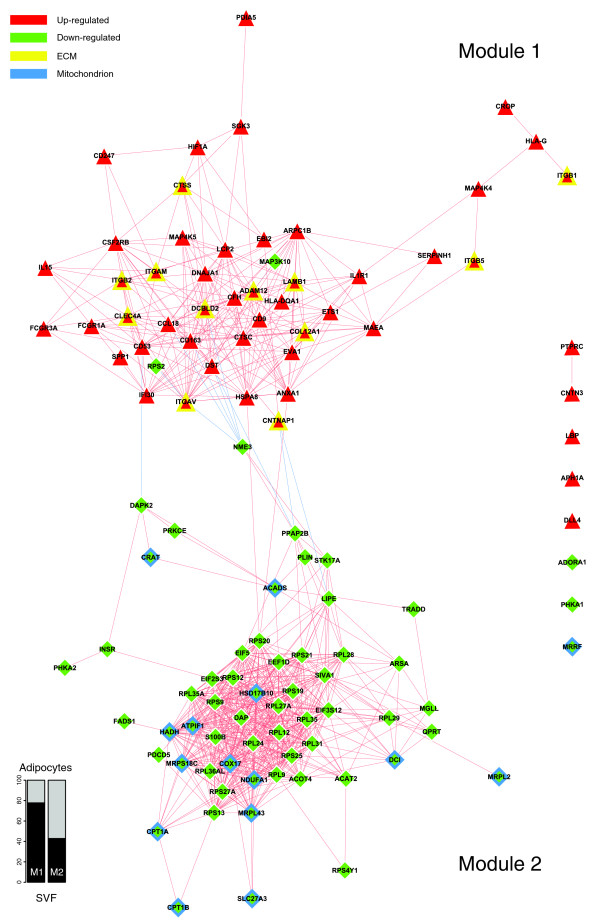
Gene co-expression network underlying the GO Biological Process interaction map in obese WAT. The relationships of differentially expressed genes annotated with over-represented categories of the GO Biological Process ontology were determined in order to build a co-expression network. The absolute value of a Spearman's correlation coefficient Rs ≥ 0.8 between expression profiles was used as a co-expression threshold to relate co- or inversely expressed genes. Red lines indicate co-expression relationships while blue lines illustrate inverse expression relationships. Genes with a yellow border code for known ECM components, while genes with a blue border are related to mitochondrial components. The enrichment in genes expressed preferentially in one of the two main cellular fractions of WAT, illustrated in a percentage scale (mature adipocytes in light gray versus SVF in black), was significantly different in the two modules (*p *value < 0.05). The shapes indicate the module to which the analyzed genes belong: a triangle for Module 1 and a lozenge for Module 2.

**Figure 5:**
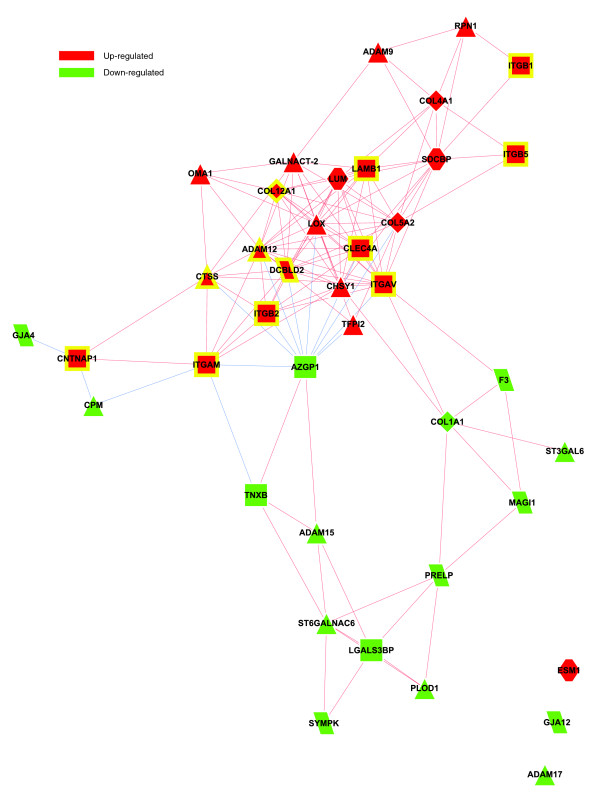
Co-expression network of ECM related genes showing significant differential expression in obese WAT. The relationships of differentially expressed genes annotated with structural or functional GO categories related to ECM were determined in order to build a co-expression network. The absolute value of a Spearman's correlation coefficient Rs ≥ 0.8 between expression profiles was used as co-expression threshold to relate co- or inversely expressed genes. Red lines indicate co-expression relationships while blue lines illustrate inverse expression relationships. Genes with a yellow border are annotated with significantly over-represented GO Biological Process categories (Figures 2 and 4). The shapes illustrate the membership of those genes in different families of ECM components among those listed in the online supplementary table 1 and the Additional file 2.

Figure 4 depicts a bi-modular co-expression network relating genes annotated with significantly over-represented GO Biological Process categories in the obese WAT (Figure 2a). The first co-expression module (Figure 4, module 1) groups up-regulated genes associated with processes constituting the first functional interaction module (Figure 2b, module 1). This module includes representatives from all major classes of ECM components, namely structural proteins such as members of the collagen family, adherent proteins such as fibronectin and laminin family members, glycosaminoglycans and proteoglycans, and specialized glycoproteins such as integrins, as well as several enzymes involved in ECM remodeling (Additional data file 2 and supplementary online table 1). A sub-network grouping all ECM related genes, showing significant differential expression in obese WAT, is presented in Figure 5.

Among various ECM components, several genes coding for members of the integrin family were found to be significantly induced and co-expressed in obese WAT, occupying central positions in the first co-expression module (Figure 4, module 1). This module included integrins alpha V (*ITGAV*), referred to as the vitronectin receptor, and alpha M (*ITGAM*), as well as integrins beta 1 (*ITGB1*; also named fibronectin receptor or beta polypeptide), beta 2 (*ITGB2*) and beta 3 (*ITGB5*). These integrins displayed strong co-expression with other key components of the ECM (Figures 4 and 5; Additional data file 2 and supplementary online table 1), such as members of the collagen family, including the major type IV alpha collagen chain of basement membranes (*COL4A1*), and members of the fibril associated collagen (*COL5A2 *and *COL12A1*). They were also co-expressed with members of the glycosaminoglycan and proteoglycan family (syndecan binding protein (*SDCBP*), lumican (*LUM*)), known to play an important role in the initiation of inflammatory phenomena, as well as in the recruitment, rolling, and subsequent extravasation of lymphocytes [24], the laminin beta 1 (*LAMB1*), and with several proteases and other enzymes involved in ECM remodeling and cell-cell or cell-matrix interactions. Some of the genes coding for these enzymes were significantly induced in the obese WAT. Among them, metalloproteinases domain 12 (*ADAM12*) and domain 9 (*ADAM9*), which belong to the disintegrin family, are known to modulate the communication between the fibronectin-rich ECM and the actin cytoskeleton, and are also involved in the early stages of pre-adipocyte differentiation [25]. Lysyl oxidase (*LOX*) is involved in cross-linking extracellular matrix proteins, while chondroitin sulfate GalNAcT-2 (*GALNACT-2*) plays a central role in the synthesis of some members of the glycosaminoglycan and proteoglycan family. Other ECM related genes were significantly under-expressed in WAT of obese subjects, such as metallopeptidases domain 17 (*ADAM17*) and domain 15 (*ADAM15*), or the collagen type I alpha 1 (*COL1A1*).

Interestingly, the first co-expression module (Figure 4, module 1) grouped not only genes related to ECM components, but also a number of genes coding for cytokines and surface markers secreted by immune cells possibly infiltrating WAT in obese subjects. A number of these genes showed significant co-expression with members of the integrin family and are known to be involved in the recruitment and activation of immune circulating cells, such as monocytes, lymphocytes or neutrophils. Among them were markers of the alternative pathway of macrophage activation, as the CC chemokine ligand 18 (*CCL18*) and the macrophage scavenger receptor (*CD163*), which showed strong co-expression with the integrin alpha V (*ITGAV*) and the macrophage receptor 1 (*Mac-1*) complex formed by integrins alpha M (*ITGAM*) and beta 2 (*ITGB2*). Available data demonstrate that the synthesis of CCL18 by alternatively activated macrophages is induced by Th2 cytokines, integrin beta 2 (*ITGB2*) and the scavenger receptor (*CD163*) [26]. CCL18 is also known to be involved in the recruitment and activation of CD4+ and CD8+ T cells and, more remarkably, is credited with playing a central role in perpetuating fibrotic processes through its involvement in a positive feedback loop that links activated macrophages to fibroblasts [26]. Moreover, expression of the Mac-1 complex is increased by conditions such as diabetes, being overweight and tissular hypoxia [27,28], and plays an important role in the recruitment, adhesion, and activation of circulating monocytes and neutrophils, and in the phagocytosis of complement coated particles [28,29]. Co-expressed with Mac-1 components, the hypoxia-inducible factor 1 (*HIF1A*) is a well characterized transcription factor that performs an essential role in cellular responses to hypoxia. HIF1A is also involved in the regulation of macrophage migration, and modulates the metabolism of immune cells exposed to low oxygen tensions in hypoxic areas of inflamed tissues [30].

To the same group of pro-inflammatory molecules belong also interleukin (IL)1 receptor type I (*IL1R1*), which modulates many cytokine induced immune and inflammatory responses, and IL15 (*IL15*), which regulates T and natural killer cell activation and proliferation [31,32]. Both of them were strongly co-expressed with the Mac-1 complex and with C-type lectin domain family 4 member A (*CLEC4A*), known to play an important role in mediating the immune and inflammatory responses, especially in neutrophils [33].

Several molecules demonstrated strong co-expression with IL1R1, among which are the CD53 (*CD53*) and CD9 (*CD9*) markers, known to complex with integrins, and annexin I (*ANXA1*), credited with a potential anti-inflammatory activity, all of them performing important homeostatic roles by modulating innate immunity [34-36]. In the same spectrum, integrin alpha V (*ITGAV*) displayed strong co-expression with CD163, a well known macrophage-specific marker mediating an anti-inflammatory pathway that includes IL10, and whose synthesis was shown to be well correlated with local and systemic inflammatory phenomena [37,38]. Also, the heat shock protein 8 (*HSPA8*), a surface marker for the undifferentiated cellular state expressed on the surface of human embryonic stem cells [39], performs an important role in the repair processes following harmful tissular assaults (for example, hemorrhage or local ischemia) [40], and was found to be significantly co-expressed with integrin alpha V, annexin I and other ECM components.

**Figure 6:**
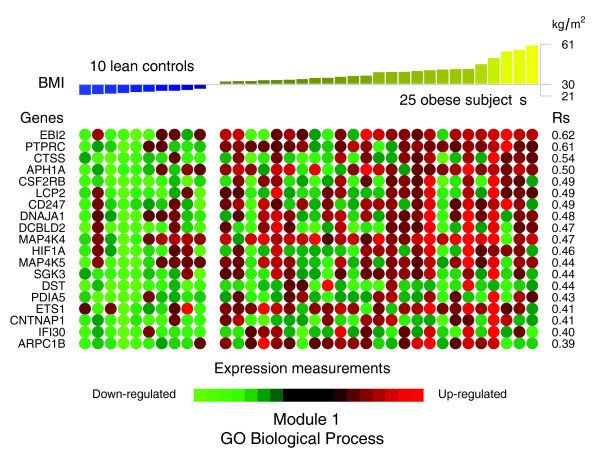
Significant correlations between the BMI and the expression profiles of the genes annotated with themes composing the first GO Biological Process interaction module in obese WAT. Significant Spearman's rank correlations between BMI and the WAT expression profiles of the genes annotated with themes composing the first interaction module (GO Biological Process) were selected in relation to a 5% FDR. The expression levels of these genes in each of the analyzed subjects are represented as green (down-regulated) or red (up-regulated) dots.

A panel of the genes clustered in module 1 of the co-expression network (Figure 4) displayed significant positive correlations between their expression levels in WAT of obese and non-obese subjects and the BMI of these subjects (Figure 6 and Table 2). Among the genes showing the strongest association with the BMI were cathepsin S (*CTSS*), involved in the degradation of several components of the extracellular matrix [19], lymphocyte cytosolic protein 2 (*LCP2*) and CD247 (*CD247*), both related to T cell development and activation, as well as the hypoxia-inducible factor 1 (*HIF1A*).

**Table 2:** Significant correlations between BMI and expression profiles of genes annotated with themes composing the GO Biological Process interaction modules in obese WAT

EntrezGene gene ID	Gene symbol	Gene name	Fold*	Rs^†^	FDR^‡^	Tissular fraction^§^
**Module 1**						
1880	*EBI2*	*Epstein-Barr virus induced gene 2 *(*lymphocyte-specific G protein-coupled receptor*)	1.63	0.62	0.00	SVF
5788	*PTPRC*	*protein tyrosine phosphatase*,*receptor type*,*C*	1.74	0.61	0.00	SVF
1520	*CTSS*	*cathepsin S*	1.53	0.54	0.00	SVF
51107	*APH1A*	*anterior pharynx defective 1 homolog A*	1.40	0.50	0.01	SVF
1439	*CSF2RB*	*colony stimulating factor 2 receptor*,*beta*,*low-affinity *(*granulocyte-macrophage*)	2.03	0.49	0.01	SVF
3937	*LCP2*	*lymphocyte cytosolic protein 2 *(*SH2 domain containing leukocyte protein of 76 kDa*)	1.87	0.49	0.01	SVF
919	*CD247*	*CD247 molecule*	1.36	0.49	0.01	SVF
3301	*DNAJA1*	*DnaJ *(*Hsp40*)*homolog*,*subfamily A*,*member 1*	1.46	0.48	0.02	SVF
131566	*DCBLD2*	*discoidin*,*CUB and LCCL domain containing 2*	2.00	0.47	0.02	-
9448	*MAP4K4*	*mitogen-activated protein kinase kinase kinase kinase 4*	1.40	0.47	0.02	-
3091	*HIF1A*	*hypoxia-inducible factor 1*,*alpha subunit *(*basic helix-loop-helix transcription factor*)	1.32	0.46	0.02	SVF
11183	*MAP4K5*	*mitogen-activated protein kinase kinase kinase kinase 5*	1.35	0.44	0.03	A
23678	*SGK3*	*serum*/*glucocorticoid regulated kinase family*,*member 3*	1.48	0.44	0.03	-
667	*DST*	*dystonin*	1.66	0.44	0.03	SVF
10954	*PDIA5*	*protein disulfide isomerase family A*,*member 5*	1.34	0.43	0.03	-
2113	*ETS1*	*v-ets erythroblastosis virus E26 oncogene homolog 1*	1.65	0.41	0.03	-
8506	*CNTNAP1*	*contactin associated protein 1*	1.24	0.41	0.03	SVF
10437	*IFI30*	*interferon*,*gamma-inducible protein 30*	2.35	0.40	0.04	SVF
10095	*ARPC1B*	*actin related protein 2*/*3 complex*,*subunit 1B*,*41 kDa*	1.74	0.39	0.04	SVF
**Module 2**						
5256	*PHKA2*	*phosphorylase kinase*,*alpha 2*	0.67	-0.67	0.00	A
3643	*INSR*	*insulin receptor*	0.67	-0.66	0.00	-
23604	*DAPK2*	*death-associated protein kinase 2*	0.44	-0.61	0.00	A
1384	*CRAT*	*carnitine acetyltransferase*	0.62	-0.61	0.00	A
1968	*EIF2S3*	*eukaryotic translation initiation factor 2*,*subunit 3 gamma*,*52 kDa*	0.63	-0.58	0.00	-
11000	*SLC27A3*	*solute carrier family 27 *(*fatty acid transporter*),*member 3*	0.76	-0.56	0.01	SVF
10572	*SIVA1*	*CD27-binding *(*Siva*)*protein*	0.69	-0.54	0.01	A
6158	*RPL28*	*ribosomal protein L28*	0.66	-0.53	0.01	SVF
6206	*RPS12*	*ribosomal protein S12*	0.53	-0.52	0.01	SVF
11224	*RPL35*	*ribosomal protein L35*	0.65	-0.51	0.01	SVF
6187	*RPS2*	*ribosomal protein S2*	0.77	-0.51	0.01	-
51069	*MRPL2*	*mitochondrial ribosomal protein L2*	0.59	-0.50	0.01	A
93974	*ATPIF1*	*ATPase inhibitory factor 1*	0.79	-0.49	0.01	-
134	*ADORA1*	*adenosine A1 receptor*	0.75	-0.48	0.01	A
51023	*MRPS18C*	*mitochondrial ribosomal protein S18C*	0.74	-0.48	0.01	A
35	*ACADS*	*acyl-CoA dehydrogenase*,*C-2 to C-3 short chain*	0.69	-0.48	0.01	A
6227	*RPS21*	*ribosomal protein S21*	0.62	-0.48	0.02	SVF
4694	*NDUFA1*	*NADH dehydrogenase *(*ubiquinone*)*1 alpha subcomplex*,*1*,*7.5 kDa*	0.86	-0.48	0.02	A
1936	*EEF1D*	*eukaryotic translation elongation factor 1 delta *(*guanine nucleotide exchange protein*)	0.75	-0.47	0.02	SVF
3991	*LIPE*	*lipase*,*hormone-sensitive*	0.76	-0.46	0.02	A
10063	*COX17*	*COX17 cytochrome c oxidase assembly homolog*	0.71	-0.45	0.02	-
5346	*PLIN*	*perilipin*	0.71	-0.45	0.02	A
27335	*EIF3S12*	*eukaryotic translation initiation factor 3*,*subunit 12*	0.73	-0.43	0.03	A
84545	*MRPL43*	*mitochondrial ribosomal protein L43*	0.78	-0.43	0.03	A
8613	*PPAP2B*	*phosphatidic acid phosphatase type 2B*	0.50	-0.42	0.03	SVF
1983	*EIF5*	*eukaryotic translation initiation factor 5*	0.76	-0.42	0.03	SVF
1611	*DAP*	*death-associated protein*	0.70	-0.40	0.04	SVF
6166	*RPL36AL*	*ribosomal protein L36a-like*	0.73	-0.40	0.04	-
6152	*RPL24*	*ribosomal protein L24*	0.74	-0.40	0.04	SVF
122970	*ACOT4*	*acyl-CoA thioesterase 4*	0.65	-0.40	0.04	A
5255	*PHKA1*	*phosphorylase kinase*,*alpha 1*	0.81	-0.39	0.04	-
6165	*RPL35A*	*ribosomal protein L35a*	0.72	-0.39	0.04	SVF

*Gene expression fold change in the obese versus lean condition. ^†^Spearman's correlation coefficients between gene expression profiles and the BMI of analyzed subjects. ^‡^The q-values obtained by applying the Storey (2002) FDR method to adjust the *p *values computed with the Spearman's correlation test. ^§^Genes expressed predominantly in one of the two main cellular fractions of the adipose tissue: mature adipocytes (A) or the stroma vascular fraction (SVF). Hyphens indicate genes for which no significantly predominant expression in one of the two main cellular fractions of the adipose tissue could be detected.

#### The adipose metabolism related genes

The second co-expression module (Figure 4, module 2) grouped several genes encoding proteins involved in lipolysis pathways, which were down-regulated in the obese WAT, including hormone-sensitive lipase (*LIPE*), perilipin (*PLIN*), and monoglyceride lipase (*MGLL*). The insulin receptor (*INSR*) and antilipolytic adenosine A1 receptor (*ADORA1*) were also located in this module, together with a number of genes encoding mitochondrial enzymes, including NADH dehydrogenase 1 alpha subcomplex (*NDUFA1*) and cytochrome c oxidase assembly homolog (*COX17*). The *NDUFA1 *gene encodes a component of respiratory chain complex I that transfers electrons from NADH to ubiquinone, while COX17 might contribute in the mitochondrial terminal complex to the functioning of cytochrome c oxidase, which catalyzes electron transfer from the reduced cytochrome c to oxygen. Several genes of module 2 (Figure 4; online supplementary data [20]) are involved in the synthesis, transport and oxidation of a variety of fatty acids. Among them, some genes are known to code for proteins intervening in the initial step (acyl-coenzyme A dehydrogenase (*ACADS*)), and the processing (3-hydroxyacyl-CoA dehydrogenase type II (*HADH*), 3,2 trans-enoyl-CoA isomerase (*DC1*)) and the termination (acyl-CoA thioesterase 4 (*ACOT4*)) of the mitochondrial fatty acid β-oxidation pathway. The β-oxidation of long-chain fatty acids usually implicates the sequential action of carnitine palmitoyltransferase I and carnitine palmitoyltransferase II together with a carnitine-acylcarnitine translocase. The expression levels of two members of the carnitine/choline acetyltransferase family (*CPT1A *and *CPT1B*) involved in this rate limiting step across the mitochondrial inner membrane were decreased as well as that of the *CRAT *gene, which catalyzes the reversible transfer of acyl groups from an acyl-CoA thioester to carnitine and regulates the ratio of acylCoA/CoA in the mitochondrial compartments. Interestingly, module 2 also gathered several genes involved in the induction of apoptosis, such as the death-associated protein (*DAP*), the death-associated protein kinase 2 (*DAPK2*), and the serine/threonine kinase 17a (*STK17A*), a member of the DAP kinase-related apoptosis-inducing protein kinase family, as well as the apoptosis-inducing factor (*SIVA1*), TNFRSF1A-associated via death domain (*TRADD*) and programmed cell death 5 (*PDCD5*), some being strongly co-expressed with mitochondrial enzymes described above. Protein kinase C epsilon (*PRKCE*), involved in several intracellular signaling pathways and particularly in apoptosis, was linked to *DAPK2*, *CRAT*, and *ACADS *in this module. Other down-regulated genes encode components of cytoplasmic or mitochondrial ribosomal subunits, which are part of ribosomal proteins, and several eukaryotic translation elongation factors implicated in protein synthesis.

**Figure 7:**
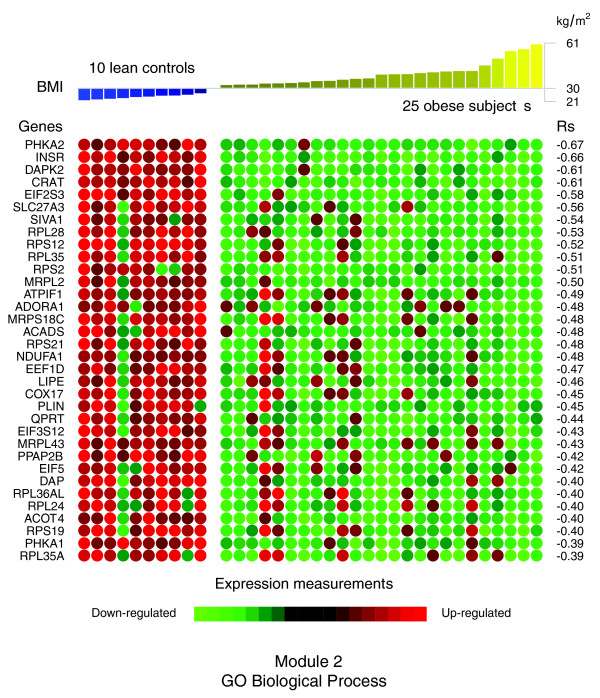
Significant correlations between the BMI and the expression profiles of the genes annotated with themes composing the second GO Biological Process interaction module in obese WAT. Significant Spearman's rank correlations between the BMI and the WAT expression profiles of the genes annotated with themes composing the second interaction module (GO Biological Process) were selected in relation to a 5% FDR. The expression levels of these genes in each of the analyzed subjects are represented as green (down-regulated) or red (up-regulated) dots.

In contrast with the genes comprising the co-expression module 1, the expression profiles of the majority of the genes comprising module 2 demonstrated significant negative correlations with BMI (Figure 7 and Table 2). Among them, some of the strongest negative correlations were observed for the insulin receptor (*INSR*), molecules of the adipocyte lipolytic pathway (*LIPE*, *PLIN*), some mitochondrial components (*CRAT*, *ACADS*, *NDUFA1*, *COX17*), and some members of apoptotic pathways (*DAPK2*, *SIVA1*, *DAP*). Also, the expression profiles of numerous components of cytoplasmic or mitochondrial ribosomal subunits showed significant negative correlations with the BMI (*RPL28*, *RPS12*, *RPL35*, *RPS2*, and *RPS21 *among others).

Since at the functional level the processes related to immune, inflammatory and stress responses, as well as to cell adhesion and signaling (Figures 2b and 3b, module 1), displayed an opposite regulation pattern to that of the metabolic functions (Figures 2b and 3b, module 2), we examined the links that may connect these two functional modules at the gene level, and searched for which genes could play a mediating role by linking the ECM to intracellular pathways. As shown in Figure 4, some ECM related genes were co-expressed with a set of inflammatory genes (module 1), while showing a significant inverse expression pattern to that of genes belonging to the metabolic module (module 2). Among them, integrin alpha V (*ITGAV*), CD163 and CCL18, two markers of the alternative pathway of macrophage activation, heat shock protein 8 (*HSPA8*), and contactin associated protein 1 (*CNTNAP1*), involved in the activation of intracellular signaling pathways, were strongly related to several genes encoding enzymes of the lipolytic pathway, including hormone-sensitive lipase (*LIPE*) and perilipin (*PLIN*), phosphatidic acid phosphatase type 2B (*PAP2B*), a member of the lipid phosphate phosphatases family, and to genes related to apoptosis, such as death-associated protein kinase 2 (*DAPK2*) and non-metastatic cells 3 protein (*NME3*).

#### A shift in the functional profile of the WAT transcriptomic signature three months after bariatric surgery

We have shown previously that weight loss is associated with improvement in the inflammatory profile, together with regression of macrophage infiltration in WAT [11]. To better characterize the association between adipose mass variation, local inflammatory phenomena and ECM remodeling, we further examined the functional profile of the transcriptomic signature of the obese WAT after a significant weight loss induced by bariatric surgery. Ten cDNA microarray experiments were performed from subcutaneous WAT biopsies carried out in morbidly obese subjects (BMI 47.65 ± 4.4 kg/m^2^, range 42.5-57 kg/m^2^), before and three months after undergoing a laparoscopic gastric bypass [41]. The detailed clinical and biochemical parameters of these subjects were presented elsewhere [13], and are provided as online supplementary data [20]. The analysis of differential gene expression with the SAM procedure [21], performed on the cDNA measurements with signals recovered in at least 80% of the microarray experiments, detected 1,744 up- and 1,627 down-regulated genes, corresponding to a 5% FDR. Functional analysis of these genes identified 2,687 genes (1,390 up- and 1,297 down-regulated) annotated with GO categories, and 868 genes (450 up- and 418 down-regulated) annotated with KEGG categories.

**Figure 8:**
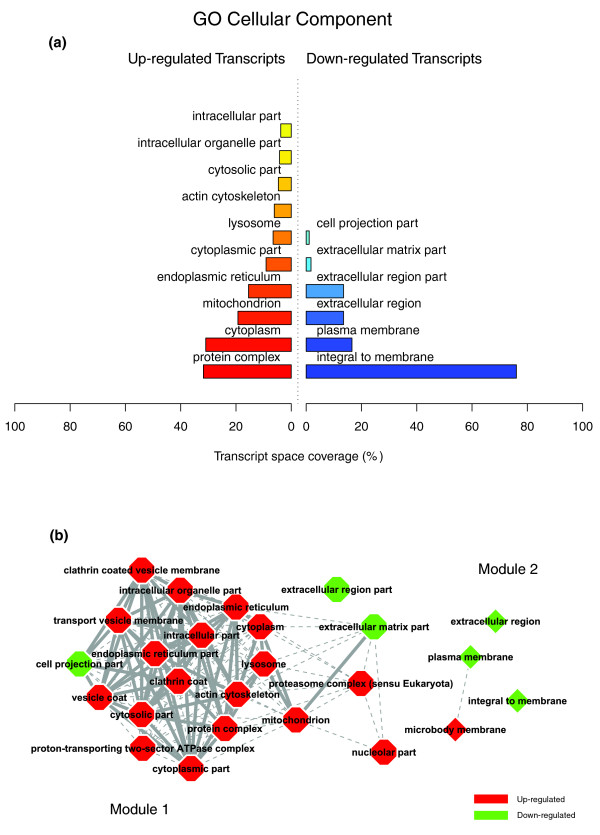
GO Cellular Component enriched themes and their interaction map, illustrating the transcriptomic signature of WAT in obese subjects three months after gastric bypass. **(a,b) **Structural themes, represented by enriched annotation categories of GO Cellular Component (a), were correlated in an interaction network after quantifying their proximity based on the expression similarity of their annotated genes (a). Continuous lines indicate the strongest interactions superior to the upper quartile of their distribution, while dashed lines depict medium strength interactions superior to the median of the distribution but inferior to its upper quartile.

**Figure 9:**
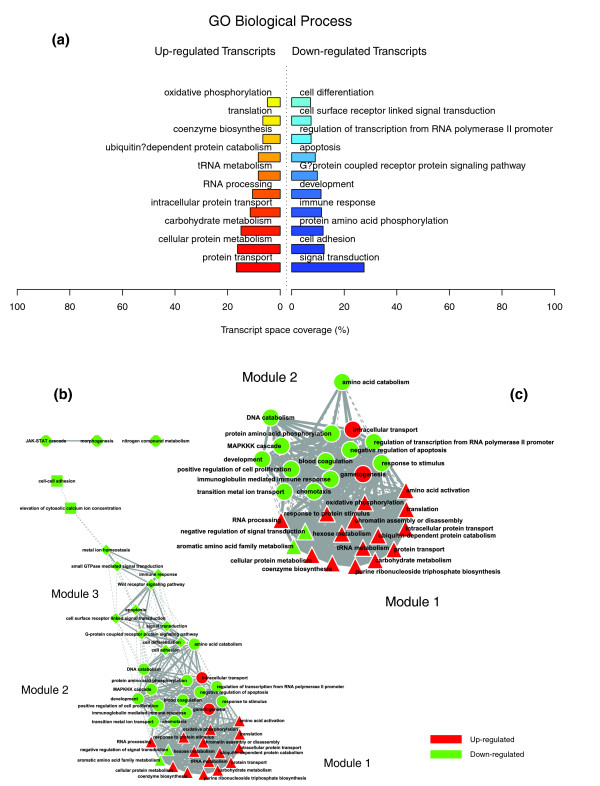
GO Biological Process enriched themes and their interaction map, illustrating the transcriptomic signature of WAT in obese subjects three months after gastric bypass. **(a,b) **Functional themes, represented by enriched annotation categories of GO Biological Process (a), were correlated in an interaction network after quantifying their proximity based on the expression similarity of their annotated genes (b). Continuous lines indicate the strongest interactions superior to the upper quartile of their distribution, while dashed lines depict medium strength interactions superior to the median of the distribution but inferior to its upper quartile. **(c) **A close-up view of the two most important functional interaction modules.

**Figure 10:**
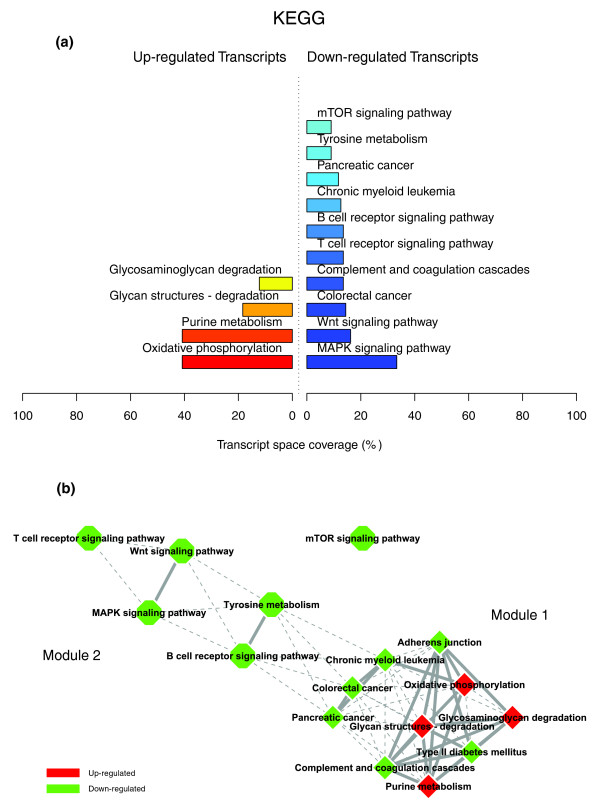
KEGG enriched themes and their interaction map, illustrating the transcriptomic signature of WAT in obese subjects three months after gastric bypass. **(a,b) **Functional themes, represented by enriched annotation categories of KEGG (a), were correlated in an interaction network after quantifying their proximity based on the expression similarity of their annotated genes (b). Continuous lines indicate the strongest interactions superior to the upper quartile of their distribution, while dashed lines depict medium strength interactions superior to the median of the distribution but inferior to its upper quartile.

Figures 8a, 9a and 10a illustrate the biological themes characterizing the transcriptomic signature of the obese WAT three months after gastric surgery, as indicated by significantly over-represented categories from GO Cellular Component (Figure 8a) and GO Biological Process ontologies (Figure 9a), and from KEGG (Figure 10a). This analysis shows a diametrical shift in the functional profile of the obese WAT associated with weight loss. Indeed, the majority of the genes up-regulated in WAT after gastric bypass were associated with structural themes (GO Cellular Component) related to the intracellular domain and organelles ('protein complex', 'cytoplasm', 'mitochondrion', 'endoplasmic reticulum', 'lysosome', 'actin cytoskeleton', 'cytosolic part'), while the down-regulated genes (Figure 8a) were mostly associated with cellular membrane and extracellular space specific themes ('integral to membrane', 'plasma membrane', 'extracellular region', 'extracellular matrix part'). The cellular processes (GO Biological Process) associated with the WAT up-regulated genes (Figure 9a) were related primarily to carbohydrate and protein metabolisms, including ubiquitin-dependent protein catabolism ('cellular protein metabolism', 'carbohydrate metabolism', 'ubiquitin-dependent protein catabolism'), to energy metabolism ('oxidative phosphorylation') and to transcriptional, translational and transport processes ('RNA processing', 'tRNA metabolism', 'translation', 'protein transport'). In contrast, down-regulated genes were mainly associated with processes related to cell adhesion and signaling (Figure 9a), notably via G-protein coupled receptor proteins ('signal transduction', 'cell adhesion', 'G-protein coupled receptor protein signaling pathway', 'cell surface receptor linked signal transduction'), as well as to the immune response and apoptosis ('immune response', 'apoptosis'). Finally, the KEGG pathways involving WAT genes up-regulated after weight loss (Figure 10a) were related to energy and nucleotides metabolisms ('oxidative phosphorylation', 'purine metabolism'), as well as to the degradation of some key ECM constituents, namely the glycosaminoglycans ('glycan structures - degradation', 'glycosaminoglycan degradation'). In accordance with GO annotations, the down-regulated KEGG pathways were related mostly to signaling processes and immune and inflammatory responses (Figure 10a), including complement and coagulation cascades and signaling of T and B cell receptors ('MAPK signaling pathway', 'Wnt signaling pathway', 'Complement and coagulation cascades', 'T cell receptor signaling pathway', 'B cell receptor signaling pathway', 'mTOR signaling pathway', and so on).

The quantification of the transcriptomic interactions relating biological themes associated with various structures, processes or regulatory pathways identified a very distinct interaction pattern from that observed in the previous condition. Figures 8, 9 and 10 illustrate a very dense interaction pattern relating up- and down-regulated processes in a strongly interconnected network. Figure 9c depicts the two most representative functional interaction modules (GO Biological Process) in this condition; this illustrates the strong interactions that connect the up-regulated themes composing the first functional module, mostly related to carbohydrate, energy and protein metabolism, with the down-regulated themes grouped in the second interaction module and related essentially to immune and inflammatory responses, signaling, cellular proliferation and apoptotic processes.

Co-expression networks underlying these functional modules (see the online supplementary data [20]) confirmed the dense interaction pattern associating genes related to the ECM and inflammatory and metabolic processes. A number of ECM components showed opposite expression patterns to those noted in the previous condition, some being induced by weight loss while others were down-regulated (online supplementary Table 2 [20]). Among others, several genes coding for structural proteins were significantly down-regulated after weight loss (online supplementary Table 2 [20]), including members of the integrin family, such as integrin alpha V (*ITGAV*), integrin beta 4 (*ITGB4*), and integrin beta 6 (*ITGB6*). Enzymes involved in the degradation of glycosaminoglycans and proteoglycans were also significantly up-regulated after weight loss, as shown by the induction of the related KEGG pathways (online supplementary data [20]). In addition, some metallopeptidases implicated in the degradation of other ECM components were equally induced, such as the matrix metallopeptidase 2 (*MMP2*), concomitantly with several metallopeptidase inhibitors from the tissue inhibitor of metalloproteinase family (*TIMP1*, *TIMP2*). Finally, a remarkable number of genes related to mitochondrial enzymes involved in the oxidative phosphorylation pathway (Figure 10; online supplementary data [20]) were significantly up-regulated after weight loss, including genes coding for NADH dehydrogenases (*NDUFA3*, *NDUFA5*, *NDUFA6*, *NDUFA9*, *NDUFA11*, *NDUFA4L2*, *NDUFB7*, *NDUFB11*, *NDUFS2*, *NDUFS8*), ATP synthases (*ATP5G1*, *ATP5G2*, *ATP5H*, *ATP5I*, *ATP5O*, *ATP6AP1*) and cytochrome c-1 (*CYC1*).

### Morphological characterization of the subcutaneous WAT in obese subjects

Analysis of functional and gene co-expression networks suggested a link between ECM remodeling, inflammatory changes and deregulation of adipocyte metabolism in relation to the degree of obesity. In chronic low-grade inflammatory diseases, prolonged inflammation stimuli result in tissue injuries that can lead to excessive synthesis of ECM elements and their progressive deposition. Examination of the functional interaction networks indicated that a similar phenomenon may occur in the obese WAT, involving the presence of inflammatory cells and a possible contribution by fibroblast derived pre-adipocytes in producing ECM components. We therefore combined series of optical, electron microscopy and immunohistochemistry analyses to examine the extracellular space of obese WAT and to quantify fibrosis in WAT of lean and obese subjects, in weight stable conditions and after weight loss.

#### Macrophages, lymphocytes and NK cells in adipose tissue of massively obese subjects

**Figure 11:**
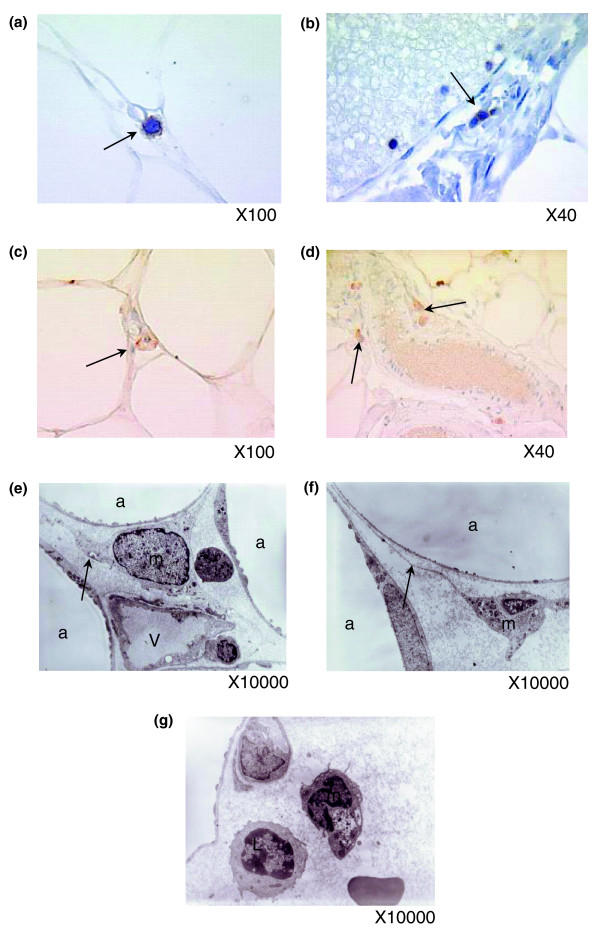
Presence of macrophages, T lymphocytes and NK cells in the subcutaneous WAT of morbidly obese subjects. **(a-d) **Immunohistochemistry on paraffin-embedded adipose tissue and nuclei staining with haematoxylin (blue) shows CD3 positive cells between adipocytes (f) and in vessel walls (b), as well as NK cells (anti NKp46) (c,d). **(e-g) **Electron microscopy of adipose tissue shows macrophages ('m') with cytoplasmic expansions (arrows) in stromal areas between adipocytes ('a') and sometimes close to lymphocytes ('L'). (a,b) Representative images of ten independent slides taken from ten obese or ten lean patients; (c,d) representative images of five slides taken from five patients.

Functional analysis using KEGG annotations showed that the pathway of NK cell mediated cytotoxicity was significantly enriched in genes up-regulated in obese WAT (Figure 3a), while the T cell receptor signaling pathway was enriched in genes down-regulated after gastric bypass (Figure 10a). Immunostaining for T lymphocytes and NK cells using CD3 and NKp46 antibodies confirmed the presence of these cells in the adipose tissue of morbidly obese subjects (Figure 11a-d), although at low abundance. Macrophages, demonstrating cytoplasmic extensions, and lymphocytes were detected by electron microscopy in the vicinity of adipocytes and near vessel walls (Figure 11e-g).

#### Increased fibrosis in the obese adipose tissue

**Figure 12:**
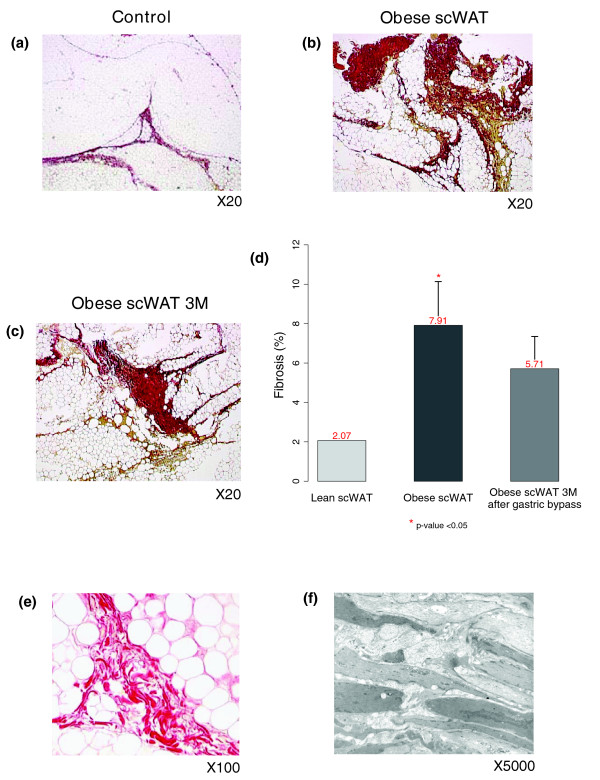
Quantification and characterization of interstitial fibrosis in subcutaneous WAT (scWAT) of morbidly obese subjects. **(a-c) **Low magnification pictures of adipose tissue connective areas (stained with picrosirius, red) in a lean control and an obese patient before and three months after bariatric surgery. Pictures are representative of ten analyzed subjects. **(d) **Automated software quantification of picrosirius areas. Error bars indicate the upper limit of the 95% confidence interval of the mean percentage of fibrosis **(e,f) **Appearance of stromal connective tissue at higher magnification (e) and by electron microscopy (f) showing layer shaped fibers.

We quantified fibrosis in the WAT of ten morbidly obese subjects before and three months after undergoing bariatric surgery, and ten age-matched lean controls (Figure 12a-d). The percentage of fibrosis in the subcutaneous WAT was significantly increased in obese subjects compared to lean controls (6.29% ± 2 versus 2.19% ± 0.25, *p *value < 0.05; Figure 12a,b,d), and remained high three months after bariatric surgery (5.7% ± 1.63; Figure 12b-d). Examination of WAT fibrotic zones in obese subjects revealed areas of swirling picrosirius stained fibers distributed in between adipocyte lobules (Figure 12e). Electron microscopy study of a similar fibrotic region showed layers of cell-free amorphous structures characteristic of extracellular matrix (Figure 12f). Additionally, we scored liver fibrosis in the same obese subjects and analyzed its relation to the amount of fibrosis in the WAT. This analysis showed that patients having the highest hepatic fibrosis score (fibrosis = 2) have also more WAT fibrosis than those with a lower hepatic fibrosis score (fibrosis = 0 or 1) (*p *value < 0.05).

#### Macrophage secretions promote ECM component expression and secretion by pre-adipocytes

**Figure 13:**
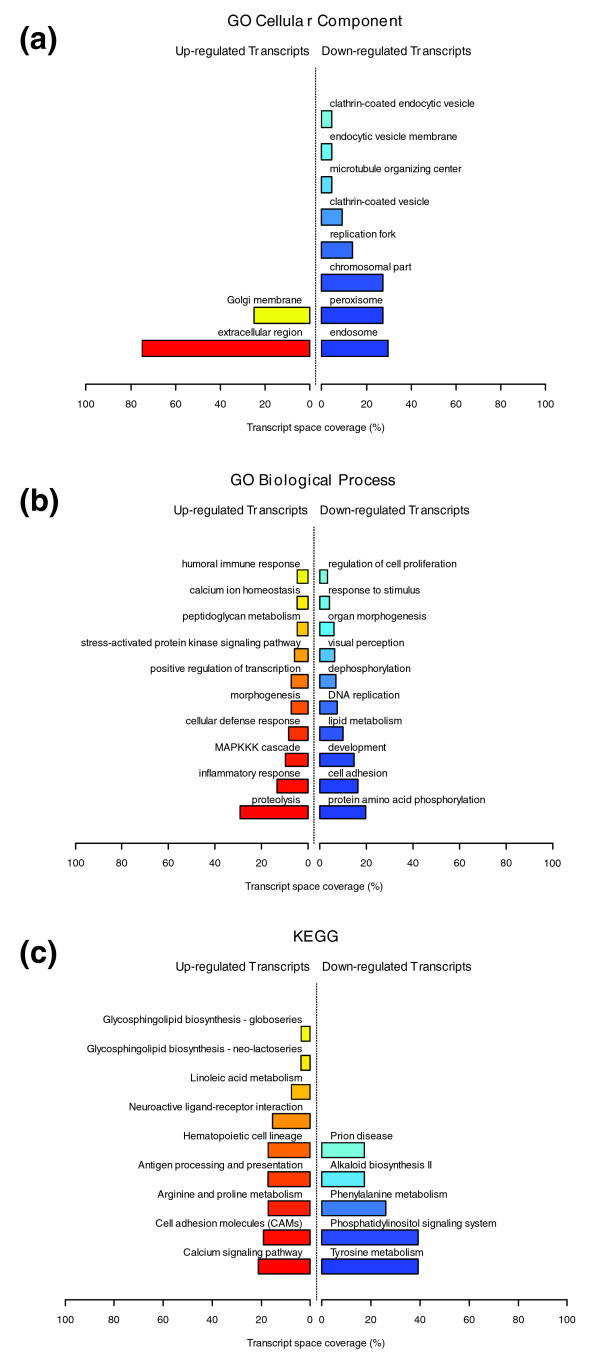
Functional profiles illustrating the transcriptomic signature of human pre-adipocytes cultivated with AcMC-conditioned media. Relevant biological themes, showing significant enrichment in genes up- or down-regulated in human pre-adipocytes cultivated with AcMC-conditioned media, are represented as annotation categories of the **(a) **GO Cellular Component and **(b) **GO Biological Process ontologies, and **(c) **KEGG.

Cellular studies were further performed to examine the possibility that pre-adipocytes may produce ECM components and cytokines with fibrotic properties when submitted to an inflammatory stimulus. To address this question, we used our previously described cell culture system in which human pre-adipocytes are cultured with activated macrophage (AcMC) conditioned media [42]. A transcriptomic analysis was performed on these cells to identify the genes and functions induced by this pro-inflammatory stimulus. More than 5,200 genes were significantly up-regulated in pre-adipocytes treated by AcMC medium (Additional data file 1). The functional analysis, using either GO or KEGG annotations, revealed that most over-expressed genes were involved in inflammatory, immune and stress responses, as well as in cell adhesion related processes, as shown in Figure 13. The examination of the genes grouped in these functions retrieved representatives from all classes of ECM components, such as structural proteins, including members of the collagen family and several precursors of collagen formation, adherent proteins, such as fibronectin 1 and its receptor, as well as laminin family members, glycosaminoglycans and proteoglycans (lumican (*LUM*)), and specialized glycoproteins, including several integrins. ECM remodeling enzymes (metalloproteases and hydroxylases involved in collagen synthesis and degradation), but also TIMP1, a natural inhibitor of the matrix metalloproteinases, were also induced (online supplementary Table 3 [20]). Among the ECM-related genes showing significant differential expression in the obese WAT compared to lean controls, 71.4% registered also significant expression changes in pre-adipocytes cultured with AcMC medium (Additional data file 2). Sixty percent of these ECM-related genes demonstrated a similar variation of their expression patterns in both *in vivo *and *in vitro *conditions, a proportion significantly greater (*p *value < 0.05) than the overall percentage of genes sharing similar expression patterns among those demonstrating a significant differential expression in the human and cell studies.

Additionally, we also observed in the cell culture study that a panel of inflammatory cytokines, including interleukins and their inducers (members of the interferon family), acute phase proteins (SAA), and chemokines (CCL5) and their receptors, were up-regulated (online supplementary Table 3 [20]). Among them, we noted the induction of IL13RA1, a subunit of the IL13 receptor complex reported to play a role in the internalization of IL13, and a major profibrotic protein known to induce transforming growth factor beta, and also of the IL4 receptor, which binds IL13 and IL4 and represents another well recognized profibrotic cytokine. It was indeed suggested that IL4 could be involved in the regulation of profibrotic events [43]. CCL5/rantes, known to stimulate liver fibrogenesis [43,44], was also induced. Also, real time quantitative PCR (RTqPCR) analysis of the gene encoding transforming growth factor beta in this set of experiments showed a 2.5-fold increase in pre-adipocytes treated by AcMC-conditioned media (*p *value < 0.05).

**Figure 14:**
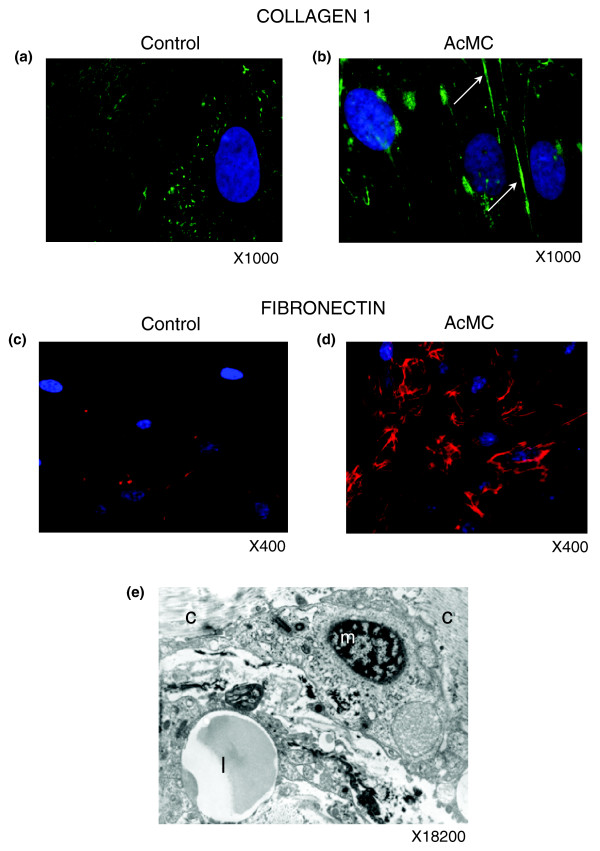
Macrophages promote ECM component secretion by pre-adipocytes. **(a-d) **Immunofluorescence analysis of pre-adipocytes cultivated with control (a,c) or AcMC-conditioned media (b,d) reveals that they express type 1 collagen (b, green staining, arrows) and fibronectin (d, red staining) in the latter condition. **(e) **Electron microscopy picture of an adipose tissue macrophage ('m') shows its collocation with collagen fibers ('c') and a lipid droplet ('l'). (a-d) Representative images of five independent experiments.

To find whether this change in gene expression pattern could be associated with an increase in the secretion of ECM proteins, we used the same cell culture system and performed immunofluorescence experiments using anti-collagen type I, the most abundant component of the ECM, and anti-fibronectin antibodies after ten days of culturing pre-adipocytes in the presence of AcMC-conditioned media. Collagen type I and fibronectin were over-expressed in AcMC-conditioned media and organized in a fiber network structure (Figure 14a-d). Electron microscopy of this ECM area illustrates macrophages in close contact with collagen type I fibers (Figure 14e).

## Discussion

### The transcriptomic signature of obese WAT illustrates the central role of ECM components in linking inflammatory and adipose metabolic anomalies

**Figure 15:**
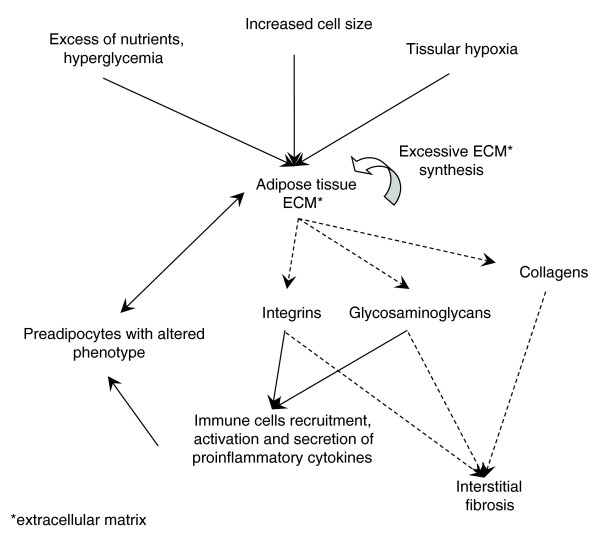
A sketch of the hypothetical map of pathophysiological interactions taking place in obese WAT. Dashed arrows indicate subcomponents of a structure (ECM) or process (fibrosis), while full arrows indicate hypothetical pathophysiological interactions.

In the present study we relied on an original strategy that combined the two conventional frameworks of functional genomic profiling and gene co-expression network analysis into an integrated analytical approach. This strategy enabled us to evaluate transcriptomic interactions between relevant functional themes and to quantify their overall significance within the global transcriptomic profile of obese WAT. The bioinformatic analysis of gene expression data identified relevant biological themes, including structural components, cellular processes and regulatory pathways, significantly enriched in up- or down-regulated genes, and compiled them into a comprehensive map of interactions illustrating the transcriptomic signature of obese WAT (Figure 15). This systematic approach provides significant advantages over conventional methods of functional profiling or transcriptomic network analysis, since it allows the extraction of robust and reliable information about the transcriptomic proximity of biological themes from the expression similarity (that is, co-expression) of their related genes. The advantage of analyzing transcriptomic interactions between biological themes is particularly well illustrated by the 'weight loss' condition, where the gene co-expression networks (online supplementary data [20]) are very dense and do not provide an immediate comprehensive view of interacting genes and related functions in the adipose tissue.

Our full-scale exploratory analysis of the obese WAT transcriptomic signature highlights the central place occupied by inflammatory and immune processes and shows the strong interaction with ECM components grouped in the same module (module 1). More precise examination of this module also suggests the involvement of several inflammatory cell types, among them T lymphocytes and NK cells, in addition to macrophages. This analysis also highlighted a segregated transcriptomic interaction pattern in obese WAT, distinguishing two interaction modules: one (module 1) grouping inflammatory and ECM related processes and another (module 2) associating adipose metabolic functions and other themes related to apoptosis and protein synthesis processes. This segregated interaction pattern was also confirmed by the observation that a significant fraction of the genes composing module 1 were positively correlated with BMI, while most of the genes grouped in module 2 showed negative correlation with the degree of obesity.

In spite of the segregated interaction pattern, the analysis of gene co-expression networks underlying the two functional interaction modules identified several candidate genes as having a mediator role in relating inflammatory phenomena and ECM remodeling to adipocyte biology. A number of up-regulated genes coding for ECM components belonging to the integrin family showed a significant inverse expression pattern with down-regulated genes coding for enzymes related to lipid and energy metabolism. It is well known that ECM modulations are transmitted to integrin complexes that regulate cytoskeleton dynamics and intracellular pathways. These phenomena have to be better understood in the context of adipocyte biology and, in particular, the links that connect ECM changes and integrin mediated signaling to processes such as cell apoptosis, protein synthesis and fatty acid oxidation in mitochondria. This latter process appeared recently to be more important than initially thought in human WAT [45].

Interestingly, the surgery induced weight loss was associated with a major shift of the WAT regulatory and interaction patterns, which reversed the functional genomic profile of the obese WAT and dramatically increased the intensity of the interactions between up-regulated adipose metabolic processes and down-regulated inflammatory and immune responses. Associated with the down-regulation of genes coding for inflammation mediators, an important number of genes related to oxidative phosphorylation and various other mitochondrial enzymes, as well as genes coding for enzymes involved in the degradation of glycosaminoglycans and proteoglycans, registered a significantly increased expression after weight loss.

### Inflammatory cells in human adipose tissue

Our analytical strategy raises several pathophysiological hypotheses that propose that an excessive synthesis of ECM components plays a mechanistic role in the constellation of anomalies characterizing obese WAT. The functional themes grouped in module 1 are enriched in genes expressed predominantly in the SVF, suggesting that several immune cell types may provide a local chronic inflammatory stimulus. Among them, we confirmed the significant presence of macrophage cells in human WAT [46]. In obese mice, a shift in the activation state of WAT macrophages from an M2 'alternatively activated' state to an M1 'pro-inflammatory state' was observed in response to diet-induced obesity [47]. The precise phenotype of macrophages in the human WAT is still unknown. Our analysis, showing the up-regulation of several genes known to be induced by Th2 cytokines, such as *CCL18 *and *CD163*, suggests that M2-polarized macrophages infiltrate the WAT of severely obese subjects. This may be associated with the presence of M1 macrophages, since genes encoding pro-inflammatory factors were also induced.

In addition to macrophages, several other lymphoid cells may synthesize families of cytokines, promoting a local inflammatory state and, thus, affecting the fibrotic response. Several genes of module 1, known to be markers of lymphocytes and NK cell activation, were strongly co-expressed with ECM components. We observed the presence of NK and T lymphocytes in obese WAT, although they appeared to be less abundant than macrophage cells. NK and natural killer T cells (NKT), as well as subclasses of T lymphocytes, have been previously described in obese WAT in animal models. A relationship between lymphocyte count and the weight of visceral and subcutaneous fat pads was also noted [48]. To date, only a few comparative studies have described the lymphoid accumulation in WAT of obese subjects [49].

### Interstitial fibrosis in human adipose tissue

Fibrosis, studied in several common diseases [50-54], is usually defined by the modification of the amount and the composition of a wide panel of ECM proteins, including collagen types (notably fibrillar collagens I and III) and glycoproteins (laminin, fibronectin, elastins). The persistence of tissue injuries can lead over time to an excessive production of ECM components, which accumulate progressively and may result eventually in impaired tissular function. Both our functional analysis and cellular studies indicate that such a pathological process might occur in obese WAT. Histological examination confirmed that the subcutaneous WAT of obese subjects had a significant increase of interstitial fibrosis, as suggested previously by a more limited assessment performed in obese children [55]. The fibrotic material was located around adipocytes, forming amorphous zones in electronic microscopy, possibly indicative of tissue deterioration. Ffibrosis quantification in the same subjects three months after bariatric surgery found no significant decrease of interstitial fibrosis, in spite of a significant down-regulation of the genes related to inflammatory and immune responses and extensive variations in the expression of genes involved in ECM remodeling. One possibility is that there is a degree of irreversibility of WAT interstitial fibrosis, consistent with processes previously described in the liver [56]. The irreversibility of hepatic fibrosis has been challenged since some authors hypothesize a potential resolution step involving the activation of ECM degradation enzymes from the matrix metalloproteinase family [57]. The co-expression network analysis showed a concomitant up-regulation of genes related to both matrix metalloproteinase and tissue inhibitor of metalloproteinase families (online supplementary Table 2 [20]), which could explain the reduced degradation of ECM components after weight loss. Another possibility could be that fibrosis may take more than three months to resolve, lagging behind the amelioration of WAT inflammatory status observed after bariatric surgery. It is noteworthy to mention that some dissociation between mechanisms regulating fibrosis and inflammatory processes have also been proposed [58].

### Cell types producing ECM components in adipose tissue: the role of pre-adipocytes

In fibrotic diseases it has been shown that the accumulation of ECM components can be driven primarily by inflammatory processes [59]. Several cell types in adipose tissue may have the capacity to synthesize ECM components, particularly in a pro-inflammatory environment characterized by an excessive production of a wide panel of cytokines and chemokines, some with well-described profibrotic properties. The transcriptomic profile of the two main cellular fractions of adipose tissue (adipocytes and SVF cells) obtained from overweight human subjects [13] showed that genes encoding ECM components or related to inflammatory processes were predominantly expressed in the SVF. This observation is supported by the RTqPCR quantification of a panel of ECM related genes, performed separately in the two cellular fractions of obese and lean subjects. Indeed, this quantification showed that, for most of the analyzed genes, the increase in their expression level in the obese state occurs more predominantly in the SVF cells than in mature adipocytes [20], thus supporting the predominant role of these cells in the excessive production of ECM components affecting the adipose tissue of obese subjects. However, these results do not exclude the role of mature adipocytes in the excessive synthesis of some ECM components.

We formulated the hypothesis that pre-adipocytes in the presence of inflammatory stimuli might contribute to the synthesis of ECM components. Recent data provided by our team showed that human pre-adipocytes in contact with activated macrophage media display a fibroblastic-like appearance, significantly proliferate and acquire pro-inflammatory properties [42]. Microarray analysis confirmed that pre-adipocytes treated by AcMC-conditioned media displayed an increased expression of a panel of genes related to ECM components or involved in inflammatory processes. In agreement, human pre-adipocytes cultured in the presence of AcMCs increased their production of fibronectin and collagen type I, which formed a fibrous network around the pre-adipocytes. Whether different factors produced by other adipose SVF cells in obese subjects could contribute to modify the pre-adipocyte phenotype in a similar manner as the one observed with AcMC media needs to be further explored, as well as the participation of other cell types, such as myofibroblasts or fibroblasts derived from blood-borne mesenchymal progenitors. A more precise characterization of the cells composing the adipose SVF is necessary to determine if such cell types are also components of the human WAT.

## Conclusion

From a temporal perspective, human obesity can be considered as a set of phenotypes that develop successively over time. In this sequence one can distinguish: a 'pre-obese static phase' when the individual at risk of obesity has a stable weight and energy balance status; a 'dynamic weight gain phase' during which weight increases as a result of a positive energy balance with intakes exceeding expenditures; and an 'obese static phase' when the individual stabilizes their weight status at a higher level and the energy balance is re-established [60]. Once the obese phase is attained, the new weight status appears to be strongly defended by both biological and psychological regulatory mechanisms. In the initial phase, behavioral and environmental factors could play a key role in the constitution of adipose tissue excess on a genetically predisposed background [61]. Progressive biological alterations of adipose tissue metabolism could also lead to some degree of irreversibility and contribute to the development of obesity-linked metabolic and cardiovascular complications. As suggested by studies in mice and, to a lesser degree, in humans, inflammation characterized by the infiltration of various types of circulating immune cells appears to follow the different phases of fat mass accumulation, but the mechanisms and roles of these inflammatory phenomena in the different stages of human obesity remain to be established. Our study of functional profiles and transcriptomic interactions characterizing the adipose tissue of subjects in the obese static phase confirm the strong relationship linking inflammatory processes and ECM remodeling, associated with different inflammatory cell types and to some degree of interstitial fibrosis in WAT. Fibrosis may be more than a passive witness of the pathologic state of the tissue, possibly indicating a degree of irreversibility in the evolution of obesity, as seems to be suggested by its persistence after a drastic decrease of the adipose mass, in spite of the regression of the local inflammatory phenomena. More needs to be understood about the dynamics of WAT fibrosis in the different stages of obesity, its role in the perturbation of pre-adipocyte and adipocyte biology, and in the resistance to weight loss. Regardless of the actual mechanism explaining its persistence, the increase of interstitial fibrosis in the adipose tissue could impair cell-cell contact and, therefore, interfere with cellular signaling mechanisms that regulate adipogenesis and metabolic functions of WAT. Our work opens new perspectives on the molecular mechanisms involved in fibrosis development and its possible consequences for adipose tissue function.

## Materials and methods

### Subjects and study design

Fifty five obese subjects (BMI 44.07 ± 9.06 kg/m^2^, aged 40.13 ± 11.67 years) and 15 lean controls (BMI 23.67 ± 1.51 kg/m^2^, aged 34.2 ± 8.52 years) were prospectively recruited in the nutrition department at the Hôtel-Dieu hospital (Paris, France) between 2002 and 2006. We excluded subjects with associated acute or chronic inflammatory diseases, infection and/or cancer. All obese subjects had a stable weight status for at least three months before inclusion. Ten of the obese subjects underwent gastric bypass, which was performed in the surgery department of Hôtel-Dieu hospital (Paris, France). Clinical and biochemical parameters were assessed and recorded at their peak weight before and three months after bariatric surgery. Blood samples were withdrawn after overnight fasting for biochemical testing of circulating proteins (such as serum leptin, adiponectin, IL6, tumor necrosis factor (TNF)α and high-sensitivity C reactive protein (hsCRP)). Controls were healthy lean subjects with no personal history of obesity undergoing esthetic surgery procedures. The overall clinical and biochemical parameters of the analyzed subjects are shown in Table 1. Further details for each subgroup of subjects are provided as online supplementary data [20]. There was no significant difference in terms of age between obese subjects and lean controls. All clinical investigations were performed according to the Declaration of Helsinki and were approved by the Ethics Committees of Hôtel-Dieu hospital (Paris, France). Signed informed consents were obtained for all subjects involved in the study.

### Laboratory tests

Blood samples were collected after an overnight fast of 12 hours. Glycemia was measured by enzymatic methods. Serum insulin concentrations were measured using a commercial IRMA kit (Bi-INSULINE IRMA, CisBio International, Saclay France). Serum leptin and adiponectin were determined using a radioimmunoassay kit from Linco research (Saint Louis, MI, USA), according to the manufacturer's recommendations. The sensitivity of these assays was 0.5 ng/ml and 0.8 ng/ml for leptin and adiponectin, respectively. Serum levels of IL6 and TNFα were measured by an ultrasensitive ELISA system (QuantikineUS, R&D Systems Europe Ltd, Abingdon UK). The sensitivity of this assay was < 0.04 pg/ml and 0.12 pg/ml for IL-6 and TNFα, respectively. Intra-assay and inter-assay coefficient of variation (CV) were below 8% for IL6 and 8.8% and 16%, respectively, for TNFα. Orosomucoid and hsCRP were measured using an IMMAGE automatic immunoassay system (Beckman-Coulter, Fullerton, CA, USA). The sensitivity was 35 mg/dl and 0.02 mg/dl, respectively. Intra-assay and inter-assay CV were below 4% and 6%, respectively, for orosomucoid and below 5% and 7.5%, respectively, for hsCRP.

Insulin sensitivity of subjects was evaluated using the quantitative insulin sensitivity check index (QUICKI) method, which was shown to be well correlated with the hyperinsulinemic euglycemic clamp method, considered as the reference method. The calculation was performed for fasting glucose and insulin as described previously [62].

### Microarray experiments

Samples of subcutaneous WAT were obtained from the peri-umbilical region of obese and lean subjects through a needle aspiration procedure. Total RNA was prepared using the RNeasy total RNA Mini kit (Qiagen, Courtaboeuf, France), according to the manufacturer's protocol. The concentration of total RNA was determined using a Ultrospec 2000 spectrophotometer (Pharmacia Biotech, Piscataway, NJ, USA) and the integrity of the RNA was assessed using a 2100 Bioanalyzer (Agilent Technologies, Massy, France). One microgram of total RNA from each sample preparation was amplified using the MessageAmp RNA kit (Ambion, Austin, TX), and 3 μg of amplified RNA (aRNA) was Cy-dye labeled using the CyScribe first-strand cDNA labeling kit (Amersham Biosciences, Orsay, France) [63,64].

To compare microarrray experiments performed in obese and lean subjects, we used a common reference pool generated by mixing equal amounts of total RNA extracted from adipose tissue samples of all analyzed patients. aRNA from the reference pool was labeled with Cy3, while the aRNA from the testing samples was labeled with Cy5. A total of 35 individual cDNA microarrays were performed in this condition.

In the gastric surgery condition the aRNA extracted from each WAT sample before surgery was labeled with Cy3 dye, while the aRNA from the WAT samples obtained three months after surgery was labeled with Cy5 dye. A total of ten individual cDNA microarrays were performed for this condition.

Differences in gene expression between isolated adipocytes and SVF cells were examined from subcutaneous WAT specimens of previously described overweight subjects (women, *n *= 9, BMI 27.9 ± 6.8 kg/m^2^) [11]. This group of subjects was different from the subjects who participated in the clinical investigation protocols. We compared total RNA extracted separately from adipocytes and from SVF cells, obtained after enzymatic digestion of WAT specimens and separation of the two cellular fractions as previously described [11]. It cannot be excluded that this enzymatic digestion technique may have a potential influence on the expression profiles of separated cells, as noted previously in mouse studies [65]. In this condition, the cDNA microarray experiments were performed after pooling an equal amount of total RNA from adipocyte and from SVF cell preparations, repeated six times. aRNA from SVF cells was labeled with Cy3, whereas aRNA from adipocytes was labeled with Cy5.

Finally, eight cDNA microarray experiments were performed to evaluate gene expression changes in cultured human pre-adipocytes induced by inflammatory cytokines secreted by lipopolysaccharide-activated circulating monocytes (AcMCs). The aRNA extracted from cultured pre-adipocytes incubated with control RPMI medium was labeled with Cy3 dye, while the aRNA obtained from pre-adipocytes incubated with AcMC-conditioned media was labeled with Cy5 dye.

For all these conditions, the hybridization, washing, and scanning procedures were performed as previously described [11]. Several quality cross-checks (for total RNA quality, aRNA quality, dye incorporation efficiency, and so on) and microarray 'dye swap' experiments were also performed. The raw microarray data relating to all these conditions has been deposited in the Gene Expression Omnibus [66] public repository (accession number: GSE9157).

### Real time quantitative PCR

We validated the gene expression changes by reverse transcription and RTqPCR, performed as described in [63]. These results are presented as online supplementary data [20]. We used 18S ribosomal RNA (Ribosomal RNA Control TaqMan Assay kit, Applied Biosystems, Foster City, CA, USA) as a normalization control. The primers and TaqMan probes for mRNA were obtained from Applied Biosystems. These probes were labeled with a reporter dye (FAM) on the 5' end. The probe for 18S ribosomal RNA was labeled with the reporter dyes VIC and TAMRA on the 5' end and the 3' end, respectively. For each primer and probe pair, a standard curve was obtained using serial dilutions of human adipose tissue cDNA prior to mRNA quantification.

### Statistical analyses

A print-tip loess normalization of the microarray experiments was performed after the log-transformation of the background-corrected expression measurements, as indicated in [67]. Transcripts with significant expression changes were identified by applying the SAM procedure [21]. Significant differential expression was established by imposing a 5% FDR threshold in the SAM selection procedure for all conditions. A Wilcoxon test was further used to evaluate differential expression of the genes analyzed by RTqPCR (for example, obese versus lean, before versus after bariatric surgery). Correlations between gene expression measurements, and clinical and biochemical parameters were examined with the Spearman's rank test. In all analyses the threshold for statistical significance was considered as corresponding to a *p *value < 0.05. In all conditions in which multiple testing errors were expected, due to the high number of consecutive statistical computations, the *p *values computed from the aforementioned tests were adjusted by applying the Storey (2002) correction approach [68], corresponding to an estimated FDR of 5%. All statistical analyses were performed with the R software environment for statistical computing [69].

### Analysis of the biological interactions characterizing the transcriptomic signature of obese WAT

The integrative strategy, applied to analyze differentially regulated genes, consisted of three consecutive steps: first, identification of contextually relevant biological themes through an automated annotation of differentially regulated genes; second, quantification of the transcriptomic interactions relating relevant biological themes and construction of functional interaction maps characterizing the transcriptomic signature of obese WAT in the two analyzed clinical situations; and third, analysis of the gene co-expression networks underlying the biological interaction modules and computation of network centrality measures for related gene nodes.

#### Biological annotation of differentially regulated genes

An automated annotation procedure of differentially regulated genes in microarray experiments was performed based on the GO [22] and KEGG [23] annotating systems [70,71]. EntrezGene numbers [72] were used as a standard transcript accession system to ensure a correct over-representation analysis, as they allow one to map gene identifiers to GO or KEGG biological categories. The gene annotation procedure was applied separately for each of the three GO ontologies (Biological Process, Cellular Component, and molecular function), as well as for the KEGG categories.

To minimize the false over-representation of GO categories related to the redundant annotation of genes' roles in the GO lattice, and to ensure a homogenous degree of specificity of the extracted biological annotating information, an information driven procedure was devised and applied to compute the gene enrichment for each annotating GO category (that is, the proportion of differentially expressed genes annotated with a category in relation to all the genes annotated with it). This procedure related gene enrichment computations to information-specific levels of GO, defined with respect to the precision of the annotating information encoded in the ontological lattice. A reference level of maximum annotation specificity was first defined by considering for each GO category only its directly annotated transcripts, regardless of the category's position within the GO lattice. Then, by reference to this level, we derived a number of decreasingly specific subsequent levels by transitively reassigning annotated genes within the GO lattice (that is, by reassigning annotated genes to less specific ontological categories guided by the subsumption relations encoded in the ontological lattice). Afterwards, a gene enrichment measure was computed for each GO category, at each information-specific level resulting from stepwise reassignment of GO annotations (that is, from each GO category to its direct ontological hypernyms). A *p *value of gene enrichment significance, computed from a Fisher's exact test as previously described [13,73], allowed us to identify significantly over-represented biological themes at each information-specific level. A similar gene enrichment computation was performed for KEGG categories, except that no information-specific levels were considered in this situation, as these categories are not constrained by ontological relations. The resulting *p *values were adjusted for multiple testing errors by applying the Storey (2002) FDR correction approach [68]. After expert inspection of automated annotation results, the fourth information-specific level of GO was retained for further analysis, as it provided an optimal trade-off between the specificity of the annotating information and its biological interpretability.

#### Analysis of transcriptomic interactions between over-represented biological themes

The analysis of transcriptomic interactions aimed to extract relevant information about the complex relationships between various structural and functional themes in the human WAT (that is, structural components, cellular processes, and regulatory pathways) from the similarity of the gene expression profiles annotated with significantly enriched GO or KEGG categories related to these themes. For this purpose we relied on an analytical framework designed to explore proximity relationships between abstract 'multiple-instance' objects, corresponding to annotating categories in our context, constituted as sets of annotated gene-instances represented by their vectors of expression measurements [73,74]. A non-linear dynamic system model, relating the multiple-instance representation of GO and KEGG categories to the expression profiles of their annotated genes, was used to extract information about the proximity between the annotating categories from the expression similarity of their annotated genes (C Henegar, K. Clement and J-D. Zucker, manuscript in preparation). An unsupervised spectral technique [75] was applied to the resulting adjacency matrix, encoding proximity information, to cluster themes into biological interaction modules (C Henegar, K. Clement and J-D. Zucker, manuscript in preparation). The selection of the optimal cluster partitions relied on the Silhouette quality index, used to compare clustering results because of its good relevance to the genomic context underlined in previous evaluations [76]. Finally, the Cytoscape software environment for biomolecular interaction networks analysis [77] was used to construct comprehensive maps illustrating the interactions between biological themes characterizing the transcriptomic signature of obese WAT in the analyzed conditions.

#### Analysis of the gene co-expression networks underlying biological interactions in WAT

Co-expression networks analysis was performed to relate differentially expressed genes annotated with relevant biological themes and to evaluate their biological importance based on conventional measures of network centrality. The analytical framework used to construct networks of co-expressed genes has been described in [78]. A co-expression matrix was first computed by relying on the Spearman's correlation coefficient to quantify the similarity of gene expression profiles in the analyzed conditions. Then, an adjacency matrix was obtained by applying a *signum *function to the co-expression matrix. The *signum *adjacency function implements a 'hard' thresholding approach that relies on a predetermined threshold parameter. This threshold parameter, which represents a co-expression significance indicator, was determined by maximizing a scale-free topology criterion as previously described [78]. To assess the potential biological relevance of individual genes, network centrality measures, such as the betweenness and the intra-modular connectivity of gene nodes, were computed from the constructed co-expression networks, as previously proposed [79].

### Morphometry

Peri-umbilical subcutaneous WAT biopsies were performed by surgeons during gastric surgery interventions. Samples were fixed overnight at 4°C in 4% paraformaldehyde then processed for standard paraffin embedding. Thin sections (5 μm thick) were haematoxylin-eosin stained and analyzed by three independent observers in a blind fashion with a Zeiss 20 Axiostar Plus microscope (Zeiss, Germany). Digital images were captured by a Sony triCCD camera (Sony, France). Cell diameter was measured in 400 adipocytes per sample using PerfectImage Software (Claravision, Orsay France). A mean diameter was calculated for each WAT biopsy.

### Liver histopathology

A liver biopsy was performed in each of the ten lean subjects and ten obese patients before and after weight loss. Liver samples were formalin fixed, paraffin-embedded and routinely stained (haematoxylin and eosin, Masson's trichrome, picrosirius red and Perl's staining). Slides were coded and analyzed by a single expert pathologist blind to the identity of the biopsy. Major histopathological features were recorded and semi-quantified according to previously published criteria [80]. Fibrosis was classed as stage 0 (none), stage 1 (1a-b zone 3 perisinusoidal fibrosis only, 1c portal fibrosis only), stage 2 (zone 3 perisinusoidal fibrosis and periportal fibrosis without bridging), stage 3 (bridging fibrosis) and stage 4 (cirrhosis, probable and definite).

### Fibrosis quantification

Adipose tissue was collected from ten lean and ten obese subjects, before and 3 months after bariatric surgery and paraffin-embedded as described above. These patients were the same group used to study liver histopathology. Slides were stained with picrosirius red [81]. Briefly, the sections were incubated in 0.2% phosphomolybdic acid, 0.1% picrosirius red (direct red 80 in saturated picric acid), and 0.01 N HCl and then dried and mounted in Permount. Fibrosis analysis was performed using Alphalys platform (Histolab software, Plaisir, France) at ×100 magnification with constant color thresholds. Quantification was assessed as a percentage of red staining (fibrosis)/tissue surface ratio.

### Immunohistochemistry

Immunohistochemical detection of T lymphocytes using CD3 (Neomarker Microm, Francheville France) was performed with the avidin-biotin peroxidase (ABC) method [82]. Immunohistochemical detection of NK cells using NKp46 (RnDsystem, Lille France) was performed with EAC (Dako Cytomation, Trappes France). De-waxed sections (5 μm thick) of subcutaneous WAT were processed through the following incubation steps: first, antigen unmasking by 750 W micro-wave washing in a solution of citrate buffer (10 mM pH 6.0) three times; second, hydrogen peroxide 3% in water for 15 minutes to block endogenous peroxidase; third, Tris-buffered saline/Tween20-Casein 0.02 M solution (TBS-TC) for 10 minutes; fourth monoclonal mouse antibodies diluted 1:200 (1 h) in TBS-TC at room temperature; fifth, multilink anti-mouse biotinylated immunoglobulins (Dako Cytomation) diluted 1:200 in TBS-TC for 20 minutes; sixth, the standard streptavidin-biotin-peroxidase complex (SABC) method was applied using a commercially available kit (ABCYS, Biospa, Milano, Italy); and seventh, the staining was visualized using diaminobenzidine, and the slides were counterstained with Mayer's haematoxylin. Processed slide images were acquired by a microscope-camera system (Nikon, France).

### Electron microscopy

Small fragments of tissue were fixed in 4% glutaraldehyde in 0.1 M phosphate buffer, pH 7.4, for 4 h, post-fixed in 1% osmium tetroxide, and embedded in an Epon-Araldite mixture. Semi-thin sections (2 μm) were stained with toluidine blue, and thin sections were obtained with an ultratome, stained with lead citrate, and examined with a transmission electron microscope.

### Cell culture

Pre-adipocytes were isolated from the subcutaneous adipose tissue obtained from the peri-umbilical region of five healthy non-obese women undergoing elective surgery, and cultured as described in [83,84]. Briefly, minced adipose tissue was digested by collagenase treatment. The digested material was filtered and centrifuged. The resulting pellet (the SVF) was resuspended in erythrocyte lysis buffer (154 mM NH_4_Cl, 5.7 mM K_2_HPO_4_, and 0.1 mM EDTA, pH 7.0) at 250 g for 10 minutes. After washing in phosphate-buffered saline (PBS), the SVF cells were suspended in DMEM-10% fetal bovine serum and used for cell culture at passage 2 to eliminate non-pre-adipocyte cell contamination as confirmed by negative staining for macrophage markers (Ham 56 and Mac-1). Isolated pre-adipocytes were further cultured for 24 h in 1 ml of DMEM-10% fetal bovine serum at a cell density of 10^5 ^cells per well on round glass coverslips in 12-well plates. They were then incubated with (AcMC) or without (Control) 0.25 ml AcMC-conditioned media [42], and 0.75 ml of DMEM/F12 induction medium (final concentration of 50 nM insulin, 100 nM dexamethasone, 0.25 mM inhibitor 1-methyl-3-isobutylxanthine, and 100 nM rosiglitazone) for 4 days. Then, this medium was replaced by 0.25 ml of control or AcMC medium and 0.75 ml DMEM/F12 culture medium (final concentration of 50 nM insulin and 100 nM rosiglitazone). The medium was changed every two days until the tenth day. Cells were then fixed with 4% paraformaldehyde and immunostained: after permeabilization for 5 minutes in PBS, 3% bovine serum albumin and 0.1% triton (only for collagen I staining) and blocking for 1 h in PBS and 3% bovine serum albumin the cells were incubated overnight in a 1:100 dilution of either anti-fibronectin (BD Bioscience, San-Jose, CA, USA) or Collagen I (NOVUS, Littleton, CO, USA) antibody at 4°C under constant agitation. Revelation used cyanin 2 for fibronectin, cyanin 3 for type I collagen and DAPI nuclear staining. The coverslips were mounted in Fluoprep medium (Biomérieux, Narcy L'Etoile, France) and observed with an Axiovert microscope coupled to an Axiocam HR camera (Zeiss). Colors are arbitrary applied to grayscale pictures.

## Abbreviations

AcMC, activated macrophage; aRNA, amplified RNA; BMI, body mass index; CCL, CC chemokine ligand; CoA, coenzyme A; DMEM, Dulbecco's modified Eagle's medium; ECM, extracellular matrix; FDR, false discovery rate; GO, Gene Ontology; HIF, hypoxia-inducible factor; hsCRP, high-sensitivity C reactive protein; IL, interleukin; KEGG, Kyoto Encyclopedia of Genes and Genomes; NK, natural killer; NKT, natural killer T cells; PBS, phosphate-buffered saline; QUICKI, quantitative insulin sensitivity check index; RTqPCR, real time quantitative PCR; SAM, Significance analysis of microarrays; SVF, stroma vascular fraction; TBS-TC, Tris-buffered saline/Tween20-Casein, TNF, tumor necrosis factor; WAT, white adipose tissue.

## Authors' contributions

CH, JT, DaL, JDZ and KC designed the research; CH, JT, VA, DaL, IC, and KC performed the experiments; AB, VS, NV, DoL and PB were involved in subject recruitment and the assessment of subjects' clinical and biochemical parameters; CH developed the analytic algorithms and analyzed the microarray data; CH, JT, VA, MGM and KC wrote the paper. The authors declare no conflicts of interest.

## Additional data files

The following additional data are available with the online version of this paper. Additional data file 1 contains the results of the differential expression analysis performed in human pre-adipocytes cultured with AcMC medium. Additional data file 2 is a table listing ECM-related genes showing significant differential expression in obese WAT compared to lean controls, and in pre-adipocytes cultured with AcMC medium. Further details and results are available online on the companion web-site associated to this manuscript [20].

## Supplementary Material

Additional data file 1 Results of the differential expression analysis performed in human pre-adipocytes cultured with AcMC medium. Click here for file

Additional data file 2 ECM-related genes showing significant differential expression in obese WAT compared to lean controls, and in pre-adipocytes cultured with AcMC medium. Click here for file
