# Supplementary material for: Adipose tissue transcriptomic signature highlights the pathological relevance of extracellular matrix in human obesity
Source: Genome Biol. 2008 Jan 21;9(1):R14. doi: 10.1186/gb-2008-9-1-r14 (PMC2395253; doi:10.1186/gb-2008-9-1-r14)
Supplement: Additional data file 2 — ECM-related genes showing significant differential expression in obese WAT compared to lean controls, and in pre-adipocytes cultured with AcMC medium. [file gb-2008-9-1-r14-S2.pdf]

ECM-related genes demonstrating significant expression changes in the obese WAT compared to lean controls; bold rows indicates genes showing similar expression patterns in preadipocytes cultured with activated macrophages medium (AcMC). An extended version of this table, indicating significant expression changes of ECM-related genes in the three analyzed situations, is provided as online supplementary data on the companion web site associated to the manuscript.

ECM component	Gene ID	Symbol	Name	Obese/lean		AcMC		Tissular fraction	ECM role
				Fold	q-value (%)	Fold	q-value (%)		
**Structural proteins**									
	1303	COL12A1	collagen, type XII, alpha 1	2.24	1.54	0.67	0.70	—	proper matrix
	**1290**	**COL5A2**	**collagen, type V, alpha 2**	**1.79**	**1.67**	**1.64**	**0.26**	**SVF**	**proper matrix**
	1282	COL4A1	collagen, type IV, alpha 1	1.77	0.39	0.61	0.37	SVF	proper matrix
	**1277**	**COL1A1**	**collagen, type I, alpha 1**	**0.64**	**4.72**	**0.89**	**4.77**	**SVF**	**proper matrix**
**Glycoproteins**									
	**3685**	**ITGAV**	**integrin, alpha V (vitronectin receptor, alpha polypeptide, antigen CD51)**	**2.01**	**2.44**	**1.25**	**3.24**	**Adipocyte**	**cell matrix adhesion**
	3688	ITGB1	integrin, beta 1 (fibronectin receptor, beta polypeptide, antigen CD29 includes MDF2, MSK12)	1.96	0.82	—	—	Adipocyte	cell matrix adhesion
	50856	CLEC4A	C-type lectin domain family 4, member A	1.81	3.07	—	—	SVF	cell cell adhesion
	**3684**	**ITGAM**	**integrin, alpha M (complement component 3 receptor 3 subunit)**	**1.61**	**1.00**	**2.85**	**0.00**	**SVF**	**cell matrix adhesion**

**3689**	**ITGB2**	**integrin, beta 2 (complement component 3 receptor 3 and 4 subunit)**	**1.52**	**3.65**	**1.19**	**3.94**	**SVF**	**cell matrix adhesion**
3693	ITGB5	integrin, beta 5	1.48	3.65	—	—	—	cell matrix adhesion
**3912**	**LAMB1**	**laminin, beta 1**	**1.46**	**4.72**	**1.59**	**0.37**	**—**	**cell matrix adhesion**
8506	CNTNAP1	contactin associated protein 1	1.26	4.72	0.32	0.00	SVF	cell matrix adhesion
7148	TNXB	tenascin XB	0.78	1.54	—	—	SVF	cell matrix adhesion
3959	LGALS3BP	lectin, galactoside-binding, soluble, 3 binding protein	0.68	2.44	8.00	0.00	Adipocyte	cell cell adhesion
3956	LGALS1	lectin, galactoside-binding, soluble, 1 (galectin 1)	0.64	2.44	3.95	0.00	—	cell matrix adhesion
563	AZGP1	alpha-2-glycoprotein 1, zinc	0.53	0.00	—	—	Adipocyte	cell matrix adhesion
**Glycosaminoglycans and proteoglycans**								
6386	SDCBP	syndecan binding protein (syntenin)	2.48	0.28	0.55	0.01	—	cell cell adhesion
**4060**	**LUM**	**lumican**	**1.67**	**2.44**	**2.86**	**0.00**	**SVF**	**proper matrix**
**Enzymes**								
**7980**	**TFPI2**	**tissue factor pathway inhibitor 2**	**1.74**	**0.00**	**7.06**	**0.00**	**SVF**	**remodeling**
**6184**	**RPN1**	**ribophorin I**	**1.61**	**0.00**	**1.88**	**0.00**	**—**	**remodeling**
**8038**	**ADAM12**	**ADAM metallopeptidase domain 12 (meltrin**	**1.61**	**0.00**	**1.14**	**3.94**	**Adipocyte**	**remodeling**

		**alpha)**						
4015	LOX	lysyl oxidase	1.53	3.07	—	—	SVF	remodeling
**1520**	**CTSS**	**cathepsin S**	**1.49**	**3.07**	**1.80**	**0.04**	**SVF**	**remodeling**
**8974**	**P4HA2**	**procollagen-proline, 2-oxoglutarate 4-dioxygenase (proline 4-hydroxylase), alpha polypeptide II**	**1.47**	**0.00**	**5.42**	**0.00**	**—**	**remodeling**
22856	CHSY1	carbohydrate (chondroitin) synthase 1	1.44	3.65	—	—	—	remodeling
115209	OMA1	OMA1 homolog, zinc metallopeptidase (S. cerevisiae)	1.42	0.28	0.27	0.00	—	remodeling
**8754**	**ADAM9**	**ADAM metallopeptidase domain 9 (meltrin gamma)**	**1.41**	**1.00**	**1.42**	**0.50**	**SVF**	**remodeling**
55454	GALNACT	chondroitin sulfate GalNAcT-2	1.40	1.29	—	—	SVF	remodeling
6868	ADAM17	ADAM metallopeptidase domain 17 (tumor necrosis factor, alpha, converting enzyme)	0.79	2.44	1.48	3.94	SVF	remodeling
10402	ST3GAL6	ST3 beta-galactoside alpha-2,3-sialyltransferase 6	0.77	3.65	—	—	—	remodeling
30815	ST6GALN	ST6 (alpha-N-acetyl-neuraminyl-2,3-beta-galactosyl-1,3)-N-acetylgalactosaminide alpha-2,6-sialyltransferase 6	0.76	3.07	—	—	—	remodeling
5351	PLOD1	procollagen-lysine 1, 2-oxoglutarate 5-	0.76	3.07	3.49	0.00	Adipocyte	remodeling

		dioxygenase 1						
8751	ADAM15	ADAM metallopeptidase domain 15 (metargidin)	0.71	0.82	2.34	0.02	SVF	remodeling
1368	CPM	carboxypeptidase M	0.63	1.00	2.63	0.00	Adipocyte	remodeling
**Other components**								
**131566**	**DCBLD2**	**discoidin, CUB and LCCL domain containing 2**	**1.87**	**1.00**	**1.50**	**0.11**	**—**	**cell matrix adhesion**
**11082**	**ESM1**	**endothelial cell-specific molecule 1**	**1.38**	**0.28**	**2.50**	**0.01**	**—**	**proper matrix**
**9223**	**MAGI1**	**membrane associated guanylate kinase, WW and PDZ domain containing 1**	**0.76**	**1.67**	**0.55**	**0.00**	**Adipocyte**	**cell matrix adhesion**
**8189**	**SYMPK**	**symplekin**	**0.76**	**3.07**	**0.40**	**0.00**	**Adipocyte**	**cell cell adhesion**
**57165**	**GJA12**	**gap junction protein, alpha 12, 47kDa**	**0.75**	**1.00**	**0.44**	**0.00**	**SVF**	**cell cell adhesion**
2701	GJA4	gap junction protein, alpha 4, 37kDa (connexin 37)	0.74	1.00	—	—	SVF	cell cell adhesion
5549	PRELP	proline/arginine-rich end leucine-rich repeat protein	0.73	3.07	—	—	Adipocyte	cell matrix adhesion
2152	F3	coagulation factor III (thromboplastin, tissue factor)	0.72	1.54	3.51	0.00	—	remodeling

SVF = Stroma Vascular Fraction

The indicated fold variations were measured in the cDNA microarray experiments. Differential expression q-values were computed with the SAM algorithm and are expressed in percentages (%) indicating the global FDR expected in relation with each of the selected genes.
